# Alternative Polyadenylation and Nonsense-Mediated Decay Coordinately Regulate the Human *HFE* mRNA Levels

**DOI:** 10.1371/journal.pone.0035461

**Published:** 2012-04-18

**Authors:** Rute Martins, Daniela Proença, Bruno Silva, Cristina Barbosa, Ana Luísa Silva, Paula Faustino, Luísa Romão

**Affiliations:** 1 Departamento de Genética, Instituto Nacional de Saúde Dr. Ricardo Jorge, Lisboa, Portugal; 2 BioFIG - Center for Biodiversity, Functional and Integrative Genomics, Faculdade de Ciências, Universidade de Lisboa, Lisboa, Portugal; Inserm U869, France

## Abstract

Nonsense-mediated decay (NMD) is an mRNA surveillance pathway that selectively recognizes and degrades defective mRNAs carrying premature translation-termination codons. However, several studies have shown that NMD also targets physiological transcripts that encode full-length proteins, modulating their expression. Indeed, some features of physiological mRNAs can render them NMD-sensitive. Human HFE is a MHC class I protein mainly expressed in the liver that, when mutated, can cause hereditary hemochromatosis, a common genetic disorder of iron metabolism. The *HFE* gene structure comprises seven exons; although the sixth exon is 1056 base pairs (bp) long, only the first 41 bp encode for amino acids. Thus, the remaining downstream 1015 bp sequence corresponds to the *HFE* 3′ untranslated region (UTR), along with exon seven. Therefore, this 3′ UTR encompasses an exon/exon junction, a feature that can make the corresponding physiological transcript NMD-sensitive. Here, we demonstrate that in UPF1-depleted or in cycloheximide-treated HeLa and HepG2 cells the *HFE* transcripts are clearly upregulated, meaning that the physiological *HFE* mRNA is in fact an NMD-target. This role of NMD in controlling the *HFE* expression levels was further confirmed in HeLa cells transiently expressing the *HFE* human gene. Besides, we show, by 3′-RACE analysis in several human tissues that *HFE* mRNA expression results from alternative cleavage and polyadenylation at four different sites – two were previously described and two are novel polyadenylation sites: one located at exon six, which confers NMD-resistance to the corresponding transcripts, and another located at exon seven. In addition, we show that the amount of *HFE* mRNA isoforms resulting from cleavage and polyadenylation at exon seven, although present in both cell lines, is higher in HepG2 cells. These results reveal that NMD and alternative polyadenylation may act coordinately to control *HFE* mRNA levels, possibly varying its protein expression according to the physiological cellular requirements.

## Introduction

It has been estimated that one third of hereditary genetic diseases, as well as many forms of cancer, are caused by mutations that lead to the generation of transcripts bearing a premature translation-termination codon (PTC). Most of these PTC-containing mRNAs are targets for the nonsense-mediated mRNA decay (NMD) pathway [1]–[3]. NMD is an evolutionarily-conserved post-transcriptional surveillance mechanism that selectively detects and degrades transcripts bearing PTCs. PTCs or nonsense codons can either be generated by various types of germline/somatic alterations in the DNA, or originate as a result of routine errors in gene expression. Nevertheless, PTCs can also arise as a consequence of non-faulty regulated processes of the mRNA metabolism such as somatic rearrangements in the DNA, alternative splicing or utilization of alternative AUG initiation sites. In mammalian cells, NMD depends on the interaction of the translation termination complex with a dynamic multiprotein assembly, the so-called exon junction complex (EJC) [4], [5]. These protein complexes can assist to discriminate a premature translation termination event from a normal one. According to the classical model for mammalian NMD, the EJC, or a critical subset of EJC components, is deposited 20–24 nucleotides (nts) upstream of the exon-exon junction(s) during splicing and remains associated with the mRNA during its transport to the cytoplasm [6]. Translating ribosomes subsequently displace EJCs from the open reading frame (ORF) during the initial (“pioneer”) round of translation [7], [8]. However, if an mRNA contains a PTC located more than 50–54 nts upstream of at least one exon-exon junction, the ribosome will fail to displace these distal EJC(s). In this case, when the ribosome reaches the PTC, the eukaryotic translation release factors eRF1 and eRF3 at the PTC interact *in cis* with the retained EJC(s) *via* a multiprotein bridge [9]. Of central importance in this reaction is the interaction of UPF1 with the terminating complex and with the UPF2/UPF3 components of the retained EJC(s) [9]. This interaction marks the mRNA for rapid decay. Nevertheless, the recognition of a stop codon as a PTC depends on the physical distance between the PTC and the cytoplasmic poly(A)-binding protein 1 (PABPC1), as PABPC1 and UPF1 both compete for the interaction with the eRF3 at the terminating ribosome – if PABPC1 is in close proximity to the PTC, it seems to function as an NMD repressor; on the other hand, when the interaction between PABPC1 and the termination complex is not favorable, UPF1 can interact with eRF3 in the termination complex to induce NMD [10]–[14].

NMD is an important contributor to the fidelity of gene expression as it prevents translation of potentially harmful truncated proteins from faulty mRNAs. Nevertheless, it has become clear during recent years that many physiological mRNAs are also NMD substrates, indicating a role for NMD beyond mRNA quality control, as a translation-dependent post-transcriptional regulator of gene expression [15]–[17]. In effect, a group of NMD substrates includes physiological transcripts that encode functional full-length proteins, as shown in several genome-wide RNA microarray expression profile studies [15]–[17]. Actually, the comparison of mRNA levels of normal with NMD-deficient cells revealed that the expression of approximately 10% of the human transcriptome is regulated by NMD [15]–[17]. These physiological substrates have one feature in common with their pathological counterparts: they possess a translation termination codon that is, by NMD standards, conceived as premature. This applies, for example, to the termination codons of upstream ORFs, to termination codons that are introduced into an ORF as the result of somatic DNA rearrangements, alternative splicing, ribosomal frameshifting, mRNA editing or to termination codons that are followed by splice events in the 3′ untranslated region (UTR). In some cases, these features are exploited for self-regulatory mechanisms. For example, when a gene product induces the alternative splicing of its own transcript, a PTC may be introduced into its ORF or a splice junction may be generated 3′ to the termination codon, thus directing the resulting alternative transcript to NMD. Moreover, it is suspected that potentially NMD-sensitive physiological transcripts can stand at crossing points of pathways or networks and thus modulate such pathways as a whole. A common feature of all these processes is that NMD can potentially be used to adapt protein expression to the cellular physiological needs. The large and diverse repertoire of transcripts controlled by NMD reflects the significant influence of NMD on the cell metabolism and consequently in many human diseases [18].

When mutated, the human *HFE* gene may be involved in hereditary hemochromatosis, a common autosomal recessive genetic disorder of iron metabolism characterized by excessive intestinal iron absorption that leads to iron deposition in cells and subsequent dysfunction of several organs [19], [20]. HFE protein has been recognized as a key component of human iron homeostasis machinery but its precise role is still unknown. HFE is capable of forming protein complexes with both transferrin receptors 1 (TfR1) and 2 (TfR2) in the hepatocyte membrane [21], [22]. It was recently proposed that HFE is partitioned between TfR1 and TfR2, and under increasing iron concentrations, HFE should shift away from TfR1 towards TfR2, disengaging the signalling transduction pathway that leads to the induction of the iron regulatory hormone hepcidin [23], [24].

The *HFE* genomic structure is similar to other human MHC class I-like molecules. Each of the first six exons of the corresponding mRNA encode for one of the six distinct protein domains: a signal sequence, three extracellular domains (alpha1, alpha2, and alpha3) a transmembrane region, and a short cytoplasmatic tail [20]. Although the sixth exon is 1056 base pairs (bp) long, only the first 41 bp are translated to amino acids. In fact, the native translation termination codon is located at the 5′-part of this exon and the remaining downstream 1015 bp correspond to the *HFE* 3′ UTR, along with exon seven, which is 1943 bp long [20], [25]. The *HFE* transcript is about 4.2 kb long, being essentially expressed in the liver, duodenum, small intestine, spleen and heart. In addition, several additional alternative *HFE* transcripts are also present in a plethora of human tissues [20], [25]–[29]. All mRNA isoforms are expressed at low levels and *HFE* gene expression seems to be modestly influenced by changes in cellular iron status [30]–[32]. The above-mentioned alternative *HFE* transcript species have been attributed to alternative splicing events or alternative usage of two polyadenylation [poly(A)] signals located within exon seven, at 1455 and 2958 nts downstream of the stop codon [25], [28], [29]. Nonetheless, the identification of *HFE* alternative transcripts, their tissue-specificity and abundance, as well as the biological significance of the corresponding isoforms, remain to be completely clarified.

As indicated above, previously published data regarding the specific architecture of the human *HFE* mRNA have shown that it comprises seven exons, and the native translation termination codon is located in exon six at more than 50–54 nts upstream of the exon six/exon seven junction [25], [28], which is a feature that could make this transcript a physiological target for NMD. In addition, the fact that it presents a long 3′ UTR might also result in mRNA destabilization due to NMD, as it was already shown for other transcripts [12], [13]. To explore these hypotheses, we first analysed in several human tissues, the *HFE* mRNA 3′-end processing to characterize the potential alternative polyadenylation isoforms. This analysis revealed the usage of two novel alternative polyadenylation sites, located at exons six and seven, besides the two sites previously described in exon seven. Then, we demonstrated that those *HFE* isoforms specifically using poly(A) signals at exon seven are in fact physiological NMD-targets. Our data support the conclusion that both post-transcriptional mechanisms of alternative polyadenylation and NMD can coordinately regulate cellular *HFE* mRNA levels.

## Results

### Usage of two novel alternative polyadenylation sites in the human *HFE* transcripts, located at exons six and seven

**Figure 1:**
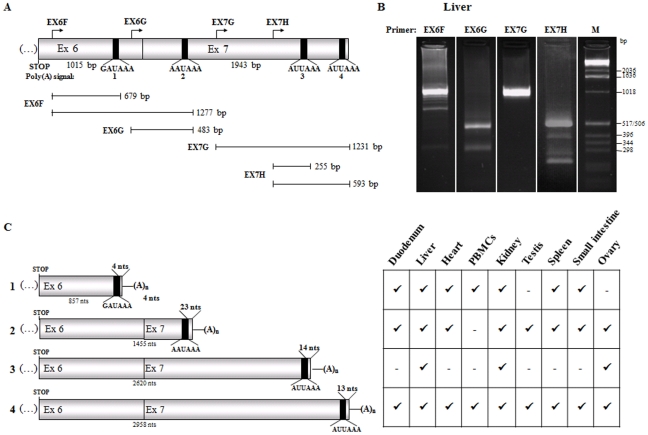
Usage of four alternative poly(A) sites for 3′-end cleavage and polyadenylation of human *HFE* transcripts. (A) The diagram represents the human *HFE* 3′ untranslated region comprising exons (Ex) six and seven. The length of the exons is shown in base pairs (bp). The position of native translation termination codon (STOP) is represented. Arrows above the diagram indicate the relative position of the four different forward primers (EX6F; EX6G; EX7G and EX7H) used in 3′-RACE experiments. The vertical black bars represent the poly(A) signals (numbered from 1 to 4) that were found to be used in the *HFE* mRNA 3′-end processing. Their sequence is also shown. Below, the thin lines represent the 3′-RACE products obtained by each primer (indicated on the left). The correspondence between each 3′-RACE product, its polyadenylation site and its length in bp is also represented. (B) Representative agarose gel electrophoresis showing 3′-RACE products from human liver total RNA, obtained by nested PCR using forward primers specified above each lane, the universal primer and the master mix provided by the BD SMART RACE cDNA Amplification Kit (BD Biosciences Clontech). The molecular weight marker (M) is the 1 kb DNA ladder (Invitrogen). (C) Schematic representation of the four human *HFE* 3′ untranslated regions identified and characterized by cloning and sequencing of the 3′-RACE products obtained from total RNA, isolated from duodenum, liver, heart, peripheral blood mononuclear cells (PBMCs), kidney, testis, spleen, small intestine and ovary. Again, vertical black bars represent the polyadenylation signal that is used in each isoform, with the corresponding sequence depicted and the distance from the poly(A) signal to the cleavage site given in nucleotides (nts). The size of each 3′ untranslated region is shown in nts, below each diagram. Each isoform is numbered from 1 to 4 according to the usage of the corresponding poly(A) signal, as depict above in *A*. (A)_n_ represents the poly(A) tail. The table on the right shows the presence (✓) or absence (−) of each *HFE* alternative polyadenylation isoform in each tissue analyzed.

Besides the two alternative polyadenylation signals, previously identified as being recognized for 3′-end cleavage and polyadenylation of the human *HFE* mRNA [25], this transcript contains several potential polyadenylation signals downstream of the native translation termination codon. To determine which poly(A) signals are in fact active in the human *HFE* transcript 3′-end processing, we carried out 3′ rapid amplification of cDNA ends (3′-RACE) experiments (Fig. 1) using nested forward primers located at *HFE* exons six and seven (Fig. 1A). This analysis was conducted in several human tissues, using total RNA from small intestine, spleen, liver, testis, ovary, duodenum, heart, kidney and peripheral blood mononuclear cells (PBMCs). After cloning and sequencing of the obtained fragments, we were able to confirm that all primers generated 3′-RACE products containing poly(A) tracts that begin 4 to 23 bp downstream of a polyadenylation signal (Fig. 1C), which are not present in genomic DNA. More specifically, a primer located at the 5′-part of exon six (primer EX6F; Fig. 1A) generated two *HFE* specific 3′-RACE products with a size of 679 and 1277 bp, respectively (Fig. 1B). These correspond to transcripts where the poly(A) signals GAUAAA and AAUAAA are recognized, respectively inducing polyadenylation at 857 and 1455 nts downstream of the stop codon [poly(A) signals 1 and 2; Fig. 1C]. The usage of the first polyadenylation signal was observed in transcripts isolated from duodenum, liver, heart, PBMCs, kidney, spleen and small intestine, whereas the usage of the second polyadenylation signal was observed in mRNA extracted from all tissues, except from PBMCs (Fig. 1C and Supplemental Fig. S1A–H). The primer located at the 3′-part of exon six (primer EX6G; Fig. 1A) allowed the detection of only one specific 3′-RACE product with a size of 483 bp (Fig. 1B). This product corresponds to transcripts in which the second poly(A) signal is recognized to induce 3′-end cleavage and polyadenylation at 1455 nts downstream of the stop codon (Fig. 1C). These results confirm those obtained with primer EX6F in what concerns the usage of the second polyadenylation signal located at 1455 nts downstream of the stop codon. Primer located at the 5′-part of exon seven (primer EX7G; Fig. 1A) generated a 3′-RACE product of 1231 bp (Fig. 1B) which corresponds to a *HFE* transcript where the poly(A) signal 4 (AUUAAA) is recognized, inducing 3′-end cleavage and polyadenylation at 2958 nts downstream of the stop codon (Fig. 1A and C). This poly(A) signal recognition was confirmed in the 3′-RACE analysis performed with primer EX7H (Fig. 1A). Here, we found two specific products with 255 and 593 bp length (Fig. 1B): the longest fragment corresponds to the recognition of poly(A) signal 4; the smallest product corresponds to the recognition of an additional AUUAAA poly(A) signal [poly(A) signal 3; Fig. 1A], which allows processing of *HFE* mRNAs with 3′-end cleavage and polyadenylation at 2620 nts downstream of the stop codon (Fig. 1B and C). Moreover, we have observed that poly(A) signal 3 is recognized in *HFE* transcripts expressed in liver, kidney and ovary (Fig. 1C and Supplemental Fig. S1A–H). On the other hand, poly(A) signal 4 is recognized in mRNAs isolated from all tissues studied (Fig. 1C and Supplemental Fig. S1A–H), which might confirm previous results showing that this signal is the main *HFE* poly(A) signal [20], [25]. In addition to the four identified and characterized 3′-RACE products, some other fragments were also obtained (Fig. 1B and Supplemental Fig. S1A–H). However, the corresponding sequencing analysis has shown that these fragments do not support *HFE* polyadenylated mRNA (File S1). These amplifications might reflect the presence of many A+T-rich sequences in the 3′ UTR *HFE* [25]. Comparing our data with previously described data [20], [25], showing the usage of two poly(A) signals at 1455 and 2958 nts downstream of the stop codon of the human *HFE* transcript, we can conclude that its 3′-end processing machinery also recognizes two novel poly(A) signals – poly(A) signals 1 and 3, respectively located at exon six and seven – which allow 3′-end cleavage and polyadenylation at 857 and 2620 nts downstream of the stop codon (Fig. 1). Although these two novel polyadenylation signals are recognized in mRNA from several tissues, the polyadenylation site 3 is used at a much lower frequency than sites 1, 2 and 4.

### The physiological human *HFE* mRNA is a natural NMD-target

**Figure 2:**
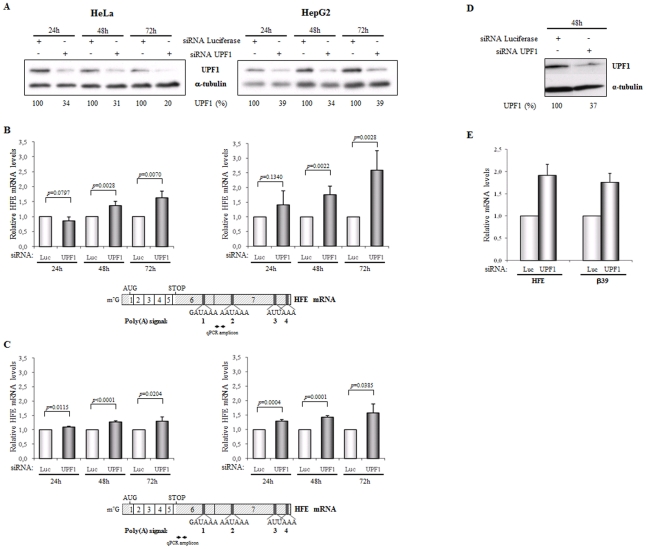
Downregulating UPF1 from HeLa or HepG2 cells results in an upregulation of the endogenous *HFE* transcripts which indicates that the physiological human *HFE* mRNA is a natural NMD-target. HeLa cells were transiently transfected with synthetic small-interfering RNA (siRNA) duplexes directed to human UPF1 or to a non-endogenous target (Luciferase; Luc) used as control. Twenty-four (24 h), forty-eight (48 h) and seventy-two hours (72 h) after siRNA treatment, cells were harvested and protein and RNA were isolated. (A) Representative Western blot analysis of the HeLa and HepG2 cells extracts transfected with human UPF1 siRNA. Immunoblotting was performed using a human UPF1 specific antibody and a α-tubulin specific antibody to control for variations in protein loading. The percentage (%) of UPF1 protein remaining expressed in the cells after siRNA treatment is indicated below each lane and was achieved by densitometric analysis using ImageJ software. (B) Relative changes in *HFE* mRNA levels were analyzed by quantitative reverse transcription-coupled real-time PCR (RT-qPCR), normalized to the levels of endogenous G protein pathway suppressor 1 (*GPS1*) mRNA. For that, cDNA was synthesized from total RNA and then, each cDNA sample was used as template for qPCR, which was performed using the SYBR Green Master Mix (Applied Biosystems) and primers that specifically hybridize to the 5′-end of *HFE* exon seven. Quantification of the transcript levels was performed by the absolute quantification method. Levels of *HFE* mRNA obtained after cellular UPF1 siRNA treatment were compared to those obtained after Luciferase siRNA treatment at the same conditions (defined as 1; arbitrary units). The histogram shows the mean and standard deviations from three independent experiments, corresponding to three independent transfections. Statistical analysis was performed using Student's *t* test (unpaired, two tailed). Below the histograms, there is a schematic representation of the human *HFE* mRNA showing its seven exons and the position of the polyadenylation [poly(A)] signals used in its 3′-end processing. The location of the initiation (AUG) and termination (STOP) codons is also represented. The double arrow represents the coordinates of the amplicon obtained in the qPCR. (C) Relative changes in *HFE* mRNA levels were quantified by RT-qPCR as in *B* but using primers that specifically hybridize to the 5′-end of the *HFE* exon six. Quantification of the transcript levels was performed by the absolute quantification method. Levels of *HFE* mRNA obtained after cellular UPF1 siRNA treatment were compared to those obtained after Luciferase siRNA treatment at the same conditions (defined as 1; arbitrary units). The histogram shows the mean and standard deviations from three independent experiments, corresponding to three independent transfections. Statistical analysis was performed as in *B*. Below the histograms, there is a schematic representation of the human *HFE* mRNA showing its seven exons and the position of the polyadenylation [poly(A)] signals used in its 3′-end processing. The location of the initiation (AUG) and termination (STOP) codons is also represented. Arrows represent the position of primers used in the qPCR. (D) Representative Western blot analysis of HeLa cells extracts transfected with human UPF1 siRNA or with a control Luciferase siRNA target. Twenty-four hours after siRNA treatment, cells were transfected with the β-globin reporter constructs (β39). Twenty-four hours post transfection, protein and RNA were isolated from the cells for analysis. Immunoblotting to confirm UPF1 knockdown was carried out with anti-UPF1 and with anti-α-tubulin antibodies (as a loading control). Identification of each band is on the right. The percentage (%) of UPF1 protein remaining expressed in the cells after siRNA treatment is indicated below each lane and was achieved as in *A*. (E) Relative β-globin mRNA levels in UPF1-depleted HeLa cells, normalized to the levels of puromycin resistance mRNA (plasmids carrying the reporter β-globin gene also contain the Puro^r^ gene), were determined by quantitative RT-qPCR and compared to the corresponding β39 mRNA levels under control conditions (Luc siRNA-treated HeLa cells) (defined as 1; arbitrary units). Using the same RNA samples, relative changes in *HFE* mRNA levels were also quantified by RT-qPCR, using experimental conditions as in *B*. Levels of *HFE* mRNA obtained after cellular UPF1 siRNA treatment were compared to those obtained after Luciferase siRNA treatment (defined as 1; arbitrary units). The histogram shows the mean and standard deviations from three independent experiments, corresponding to three independent transfections. Statistical analysis was performed as in *B*.

NMD is an mRNA surveillance mechanism that rapidly degrades mRNAs carrying PTCs [2], [3], [18], [33]. In addition to its important role in mRNA quality control, it has become clear that the NMD mechanism also plays a role in regulating the steady-state level of a set of wild-type transcripts [14]–[16], [34], [35]. These physiological NMD substrates structurally mimic nonsense transcripts as they possess a translation termination codon that is recognized as premature. In face of this knowledge, and considering the position of the human *HFE* poly(A) signals that are used for its 3′-end processing (Fig. 1), it seems evident that a percentage of the alternatively-polyadenylated human *HFE* mRNA species – those using the poly(A) signals 2, 3 or 4 (Fig. 1) – will comprise an exon/exon junction located more than 55 nts downstream of the natural stop codon, a context that can be sufficient to define the natural stop codon as a “premature stop codon” and to induce NMD. On the other hand, those transcripts resulting from cleavage and polyadenylation by usage of the poly(A) signal 1 must be NMD-resistant as no splicing event occurs downstream of the stop codon. To examine whether *HFE* transcripts could be physiological substrates for the UPF1-dependent NMD pathway, we quantified the endogenous *HFE* mRNA levels after short interfering RNA (siRNA)-mediated depletion of UPF1 in HeLa and HepG2 cells. All results were compared to those obtained in NMD-competent cells transfected with nonspecific control (Luciferase) siRNAs (Fig. 2A–C). At three different time points (twenty-four, forty-eight and seventy-two hours) after siRNAs transfection, the Western blot analysis demonstrated a decrease in UPF1 protein levels induced by siRNA of about 65–80% or 60–65%, in HeLa or HepG2 cells respectively, when compared with results obtained after treatment with Luciferase siRNAs (Fig. 2A). Under these conditions, the *HFE* mRNA levels were quantified by reverse transcription-coupled quantitative PCR (RT-qPCR) assays, relative to the *HFE* mRNA levels obtained in cells treated with the control siRNA (Luciferase siRNA). To exclusively measure the subset of *HFE* transcripts that could represent natural NMD-targets, we used oligonucleotides for qPCRs that specifically hybridize to the 5′-end of *HFE* exon seven (Fig. 2B). Our data have shown that depletion of UPF1 for seventy-two hours in HeLa cells, results in a 1.6-fold increase of the abundance of those *HFE* mRNAs using poly(A) signals 2, 3 or 4. The same analysis in HepG2 cells resulted in a 2.6-fold increase of the abundance of the same *HFE* mRNAs. These results are consistent with the mRNAs using poly(A) signals 2, 3 or 4 being natural substrates for NMD in both cell lines. However, it is interesting to note that in hepatic HepG2 cells, there is a higher amount of *HFE* NMD-targets, relatively to what occurs in HeLa cells, which might indicate that regulation of physiological levels of *HFE* mRNA by NMD is controlled in a tissue-specific manner.

To examine if the effect of the UPF1-dependent mechanism that modulates levels of *HFE* mRNA species using poly(A) signals 2, 3 or 4, is important in the context of the total amount of *HFE* mRNAs, we have also measured levels of *HFE* mRNA by RT-qPCR, using specific primers located at exon six upstream of the poly(A) signal 1 (Fig. 2C). This approach allowed quantification of all mRNAs polyadenylated at any one of the four signals. As in the previous experiment, the increase in *HFE* mRNA levels was greater after seventy-two hours of UPF1 siRNAs treatment. Under these conditions and in agreement with the preceding experiment, the expression of endogenous *HFE* mRNA, in both cell lines treated with UPF1 siRNAs, was significantly increased to about 1.3- and 1.6-fold, relative to the corresponding levels observed in cells treated with Luciferase siRNAs, in HeLa and HepG2 cells respectively (Fig. 2C). Although the amount of the total *HFE* mRNA species increased upon UPF1 siRNAs treatment (Fig. 2C), they did not reach the high levels of those *HFE* mRNAs specifically using poly(A) signal 2, 3 or 4, found to be upregulated in the previous experiment (Fig. 2B). These data corroborate the presence of the NMD-resistant *HFE* mRNA isoforms resulting from 3′-end cleavage and polyadenylation at exon six.

To confirm that the extent of UPF1 knockdown achieved in the previous experiments (Fig. 2A–C) is robust enough to reverse the effect of NMD on the expression of endogenous human *HFE* mRNA, an independent and well-characterized NMD-target transcript was analyzed at the same experimental conditions, functioning as a positive control to normalize for the extent of NMD inhibition. For that, HeLa cells were treated with Luciferase or UPF1 siRNAs as previously. Twenty-four hours later, cells were transiently transfected with a plasmid expressing the human β-globin gene carrying a nonsense mutation at codon 39 (β39), which was previously shown to induce NMD [36], [37]. Twenty-four hours later, the Western blot analysis demonstrated a decrease in UPF1 protein levels induced by siRNA to about 37%, when compared with results obtained after treatment with Luciferase siRNAs (Fig. 2D). Under these conditions, the β39 mRNA levels, as well as the endogenous *HFE* mRNA levels were quantified by RT-qPCR assays as in Fig. 2B, relative to the corresponding mRNA levels obtained in cells treated with the control Luciferase siRNA (Fig. 2E). Our data show that the UPF1 depletion resulted in an expected 1.8-fold increase in β39 mRNA levels [37]; an equivalent increase (1.9-fold increase) occurred also for *HFE* transcripts, meaning that the extent of UPF1 knockdown achieved in the previous experiments (Fig. 2A) is appropriate to analyze its impact on endogenous *HFE* mRNA expression.

**Figure 3:**
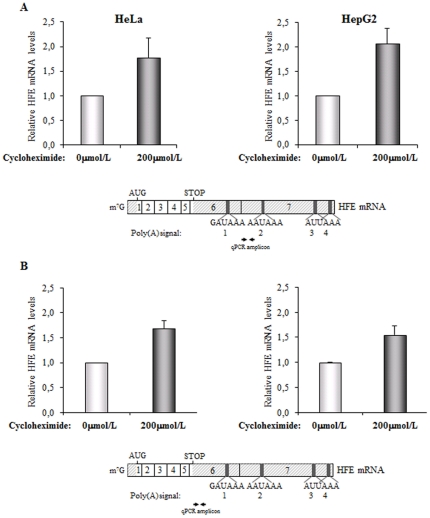
Cycloheximide treatment of HeLa or HepG2 cells results in an upregulation of the endogenous *HFE* transcripts, confirming our previous conclusion that the physiological human *HFE* mRNA is a natural NMD-target. HeLa or HepG2 cells were untreated (0 µmol/L) or treated with cycloheximide (200 µmol/L) during two hours. Then, cells were harvested and RNA was isolated. (A) Relative changes in *HFE* mRNA levels carrying polyadenylation at exon seven were analyzed by RT-qPCR, as previously in Figure 2B. Levels of *HFE* mRNA obtained after cellular cycloheximide treatment were compared to those obtained in untreated cells (defined as 1; arbitrary units). The histograms show the mean and standard deviations from three independent experiments. Below the histograms, there is a schematic representation of the human *HFE* mRNA (as in Figure 2). The double arrow represents the coordinates of the amplicon obtained in the qPCR. (B) Relative changes in total *HFE* mRNA levels were quantified by RT-qPCR as in *A* but using primers that specifically hybridize to the 5′-end of the *HFE* exon six, as shown in the diagram of the human *HFE* mRNA represented below the histograms.

To further prove by an independent assay that endogenous *HFE* mRNA is, in fact, a substrate for the NMD mechanism, and knowing that NMD mechanism is also translation-dependent, we examined the effect of inhibiting translation on *HFE* mRNA levels, by treating the HeLa and HepG2 cells with cycloheximide. *HFE* mRNA species using a poly(A) signal at exon seven were specifically quantified, as before. Results show that the expression of these endogenous *HFE* mRNA isoforms, increased to about 1.8- or 2.1-fold, respectively, in HeLa or HepG2 cell lines upon treatment with cycloheximide, relative to the levels observed in the corresponding untreated cells (Fig. 3A). Then, and to confirm the effect of NMD in the context of the total amount of *HFE* mRNA, we also measured levels of *HFE* mRNA, using the specific primers located at exon six upstream of the poly(A) signal 1 (Fig. 3B). Results have shown that after 2 hours of cycloheximide treatment, the level of total *HFE* mRNA also increases to about 1.7- or 1.6-fold relative to the levels observed in untreated HeLa or HepG2 cells, respectively. In agreement with the previous experiment performed in UPF1 knockdown cells, we observe a lower response to cycloheximide treatment when total mRNA species are quantified than when *HFE* mRNA species using a poly(A) signal at exon seven were specifically quantified.

Taken together, results from Fig. 2 and 3 demonstrate that human *HFE* mRNAs with polyadenylation at exon six are in fact expressed in HeLa and HepG2 cells and their levels contribute for the total amount of the cellular *HFE* mRNA. On the other hand, it seems that the amount of *HFE* mRNA isoforms resulting from cleavage and polyadenylation at exon seven, although present in both cell lines, is more abundant in HepG2 cells. Nevertheless, it is noteworthy that the physiological *HFE* mRNA isoforms resulting from 3′-end cleavage and polyadenylation at exon seven behave as natural targets for the UPF1- and translation-dependent NMD mechanism, both in HeLa and HepG2 cells.

### The *HFE* transcripts carrying a nonsense mutation are also committed to NMD

**Figure 4:**
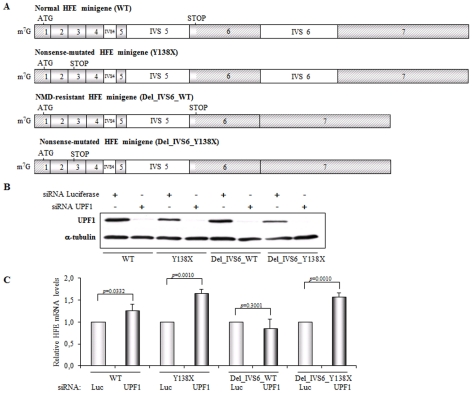
Contrary to the transcript corresponding to the minigene lacking intron 6, normal and nonsense-mutated human *HFE* transcripts are committed to NMD. (A) Schematic representation of the studied human *HFE* minigenes: normal (WT), nonsense-mutated (Y138X), lacking intron six (Del_IVS6_WT) and nonsense-mutated lacking intron six minigene (Del_IVS6_Y138X). The position of the initiation (ATG) and termination (STOP) codons is represented. The name of each minigene is indicated above each diagram. (B) Representative Western blot analysis of HeLa cells extracts transfected with human UPF1 siRNA or a control siRNA target (siRNA Luciferase). Twenty-four hours after siRNA treatment, cells were co-transfected with the plasmids encoding the above referred minigenes and with a second dose of siRNAs (UPF or Luciferase). Twenty-four hours later, cells were harvested for protein and RNA. Immunoblotting was performed using a human UPF1 specific antibody and an α-tubulin specific antibody to control for variations in protein loading. (C) *HFE* mRNA quantification was performed by RT-qPCR using primers specific for the heterologous 5′ UTR common to all transfected genes. Neomycin resistance transcript was used as a normalization control. Quantification of the transcript levels was performed by the absolute quantification method. Levels of *HFE* mRNA obtained after cellular UPF1 siRNA treatment were compared to those obtained after Luciferase siRNA treatment at the same conditions (defined as 1; arbitrary units). The histogram shows the mean and standard deviations from three independent experiments, corresponding to three independent transfections. Statistical analysis was performed using Student's *t* test (unpaired, two tailed).

The preceding study has revealed that the expression of physiological human *HFE* transcripts, specifically those species with 3′-end cleavage and polyadenylation at exon seven, are down-regulated by the NMD mechanism. To determine whether human *HFE* mRNAs carrying a nonsense mutation show a parallel NMD profile, we investigated the effect of the TAC→TAG nonsense mutation at codon 138 (Y138X) of the human *HFE* gene. This mutation had been previously described in compound heterozygosity with the C282Y mutation and associated with an iron overload phenotype in a Portuguese individual [38]. With that aim, we first cloned, into the mammalian expression pcDNA3 vector, a normal (WT) *HFE* minigene that encompasses all exons and introns 4, 5 and 6 of the human *HFE* gene (Fig. 4A). In addition, a minigene similar to the normal *HFE* construct in which intron 6 was deleted by site-directed mutagenesis (see Materials and Methods; Del_IVS6_WT minigene; Fig. 4A) was also cloned. The corresponding transcript functions as a negative control for NMD-commitment because splicing is abrogated downstream of the native translation termination codon, and thus it has the potential to be NMD-resistant. Then, by using each one of these *HFE* minigenes as template for site-directed mutagenesis (see Materials and Methods), we introduced the Y138X mutation to create the Y138X and the Del_IVS6_Y138X minigenes, respectively (Fig. 4A). In view of the higher transfection efficiency observed in HeLa cells, as compared with that obtained in HepG2 cells, the former cell line was chosen for these studies. Thus, one day after treatment with Luciferase or UPF1 siRNAs, plasmids harboring the minigenes described above were transiently transfected into HeLa cells, (see Materials and Methods). Twenty-four hours later, a Western blot analysis showed a decrease of about 95% in UPF1 protein expression induced by siRNA (Fig. 4B; lanes 2, 4, 6 and 8), when compared with results obtained after treatment with the control Luciferase siRNA (Fig. 4B; lanes 1, 3, 5 and 7). The *HFE* mRNA levels expressed in UPF1-depleted cells were determined by RT-qPCR, with specific primers for the 5′ UTR that is transcribed by the cytomegalovirus promoter of the pcDNA3 vector and is specific for all mRNAs encoded from transfected *HFE* minigenes. Expression of these genes was normalized to the expression of the neomycin resistance gene and compared to those levels of mRNA encoded by the corresponding minigene expressed in cells treated with the control siRNA (Fig. 4C). Data from three independent experiments show that cellular UPF1 depletion results in a significant 1.3-fold increase in WT mRNA when compared to its levels in control conditions (p = 0.0332), which shows that those transcripts are sensitized to NMD. In addition, when *HFE* mRNA carries a nonsense mutation at position 138 (Y138X mRNAs) and is expressed in UPF1-depleted cells, we observe a more robust effect: a 1.7-fold increase in Y138X mRNA, comparing to their expression levels in control cells (p = 0.0010). On the contrary, Del_IVS6_WT mRNA is expressed in UPF1-depleted cells at about 0.9-fold its level in control cells, showing that expression of this transcript is not significantly affected by the UPF1 depletion (p = 0.3001), behaving NMD-resistant, as expected. However, if this transcript carries a nonsense mutation at position 138 (Del_IVS6_Y138X mRNA), cellular depletion of UPF1 results in a 1.6-fold increase in Del_IVS6_Y138X mRNA levels (Fig. 4C), consistent with the blockade of the NMD pathway (p = 0.0010). Taken together, these data show that the WT, Y138X and Del_IVS6_Y138X transcripts, all are regulated by the NMD mechanism. The fact that WT transcripts show a lower increase (1.3-fold) than Y138X and Del_IVS6_Y138X (1.7 and 1.6-fold respectively) mRNAs upon UPF1 depletion, may reflect the presence of a proportion of WT *HFE* transcripts that use a poly(A) signal at exon six for 3′-end processing and thus, their NMD-resistance. On the other hand, all isoforms of Y138X and Del_IVS6_Y138X mRNAs are NMD-sensitive and thus they are all relieved from decay upon UPF1 inhibition. Alternatively, the lower levels of WT transcripts when NMD is inhibited might suggest that some other mechanism is also reducing its expression. Nevertheless, our data show that all alternatively polyadenylated mRNA isoforms encoded from an *HFE* gene that carries a PTC are down-regulated by the NMD mechanism. Physiologically, this means that only those *HFE* transcripts resulting from 3′-end cleavage and polyadenylation at exon seven behave as NMD-targets. The NMD mechanism therefore affects the levels of HFE protein.

## Discussion

The human *HFE* gene reference structure comprises seven exons [20], [25]. Although the sixth exon is 1056 bp long, only the first 41 bp encode for amino acids, thus, the remaining downstream 1015 bp sequence corresponds to the *HFE* 3′ UTR, along with exon seven. The data presented in this study show that the splicing event occurring in the 3′ UTR of the *HFE* transcript is in fact an attribute that makes the physiological transcript NMD-sensitive. In addition, we also demonstrate that besides the usage of the two polyadenylation signals for 3′-end cleavage and polyadenylation previously described [20], [25], *HFE* mRNA expression can also result from cleavage and polyadenylation at two other alternative sites – one located at exon six and the other one located at exon seven. These results illustrate that alternative polyadenylation has functional consequences, with the shorter mRNA isoform exhibiting increased stability, as they lose the feature that could make them physiological NMD-targets. Our results show that alternative polyadenylation and nonsense-mediated mRNA decay can coordinately act to control the human *HFE* mRNA levels.

HFE protein has a well-recognized role in the regulation of iron homeostasis. HFE is capable of forming protein complexes with both TfR1 and TfR2 in the cellular membrane [21], [22]. It was recently proposed that HFE is partitioned between TfR1 and TfR2, and under increasing iron concentrations, HFE should shift away from TfR1 towards TfR2, triggering the signalling transduction pathway that leads to induction of the iron regulatory hormone hepcidin [23], [24]. Here, we clearly show that, under normal conditions, human *HFE* transcript expression is affected by the NMD mechanism; in addition, a greater proportion of *HFE* transcripts are normally subject to NMD in hepatic cells. This can explain its relative low levels of expression observed in all tissues studied, including in the liver cells [29], [39]. To further corroborate the importance of this post-transcriptional mechanism in the regulation of *HFE* mRNA, there is the fact that the *TfR2* mRNA, which is thought to be the principal partner of HFE protein in the regulation of the iron metabolism in the liver, is also a natural target for NMD [16]. As a result, NMD might be used in a concerted way to adapt HFE and TfR2 protein expression to the iron physiological needs of the organism.

To our knowledge, there are few nonsense mutations reported in the human *HFE* gene [38], [40]–[42]. Yet, only one of these mutations has been studied in what concerns its ability to commit the mRNA to NMD [42]. This mutation consists in a single nucleotide deletion (c.del478) causing a frameshift that introduces a premature termination codon in exon four. The authors have demonstrated that c.del478 allele accounts for only 2% of the total cytoplasmic *HFE* mRNA showing that the mutant transcript is degraded by NMD [42]. In the light of our results, since the *HFE* mRNA containing exon seven is capable of, at some extent, escape NMD, the introduction of a nonsense mutation could lead most of the corresponding transcripts to NMD. The NMD induced by the Y138X construct was seemingly less efficient because only a 1.7-fold increase in transcript abundance was observed when subjected to UPF1 depletion. This may reflect the overexpression system in use. Moreover, it cannot be disregarded that, in the study by Pointon et al. [42], the iron overload observed in the patient could induce preferential polyadenylation at exon six and ultimately would increase levels of the wild type transcripts relatively to those of the mutated allele.

*HFE* mRNA metabolism has been poorly studied. Even the description of the specific structure of its 3′ UTR has been disputed. It was shown by Northern blot analysis that the human *HFE* gene is expressed as a 4.2 kb mRNA [20]. Nevertheless, the corresponding reported cDNA was only 2.7 kb (GenBank U60319); in fact, the remaining 1.5 kb were later described by Sanchez et al. [25] as being part of the *HFE* exon seven. Also, these authors have demonstrated by 3′-RACE experiments that human *HFE* mRNA can result from the usage of two alternative polyadenylation signals located at exon seven, at 1455 and 2958 nts downstream of the stop codon [poly(A) signals 2 and 4, Fig. 1] [25]. The observation that this 3′ UTR structure presents additional putative polyadenylation signals downstream of the native translation termination codon, led us to further investigate their possible usage for 3′-end processing. In fact, our results have shown that two novel poly(A) signals are also recognized in several human tissues. These poly(A) signals allow 3′-end cleavage and polyadenylation at exon six or seven, respectively at 857 or 2620 nts downstream of the stop codon. These poly(A) signals are recognized in mRNA from several tissues, including liver and duodenum, which are tissues where *HFE* mRNA is mainly expressed and where HFE protein has a key role in the regulation of the iron metabolism. This observation might reflect their involvement in modulating *HFE* mRNA levels in the tissues were it is specifically expressed.

Cleavage and polyadenylation is required for the maturation of most mRNA transcripts [43], [44]. Usually, the formation of mature mRNAs in vertebrates involves the cleavage and polyadenylation of the pre-mRNA at about 10–30 nts downstream of an AAUAAA or AUUAAA signal sequence. Although a strong polyadenylation signal is usually located within the 3′ UTR, in nearly all transcripts there are single-base variants of the AAUAAA sequence that can also be recognized as polyadenylation signals [45]–[48]. Large scale analyses have shown that at least ten single-base variants of the AAUAAA and AUUAAA sequences can also be found with a highly significant occurrence rate potentially representing about 22% of all polyadenylation signals [45]. In addition, Tian and colleagues have revealed that about 54% of the mRNAs display two or more polyadenylation sites [45]. In these mRNAs, the poly(A) signals proximal to the coding sequence tend to use variant signals more often, while the 3′-most sites tend to use a canonical signal [46]. Also, it has been suggested that variant signals (including the common AUUAAA) are processed less efficiently than the canonical signal and could therefore be selected for regulatory purposes [45], [46]. In the present work, we show that the human *HFE* mRNA constitutes an example of a transcript where four poly(A) signals (one AAUAAA hexamer, two AUUAAA hexamers, and one GAUAAA hexamer) can be recognized for its 3′-end cleavage and polyadenylation. These results confirm those obtained when we used the polyadq program (http://rulai.cshl.org/tools/polyadq/polyadq_form.html) [49] to evaluate potential poly(A) signals in the human *HFE* 3′ UTR – this program predicted that the AAUAAA and AUUAAA sites here described in the *HFE* exon seven [poly(A) signals 2, 3 and 4; Fig. 1] would be the active signals. It is interesting to note that in the *HFE* 3′ UTR there is the recognition of more non-canonical than canonical AAUAAA poly(A) signals, which is according to the published data [46]; in addition, the 5′-most upstream poly(A) signal is, among the four poly(A) signals that are alternatively recognized, the hexamer least frequently recognized in mammalian cells [46]. The data presented here is in fact in accordance with the usage of the four described alternative poly(A) signals to control the *HFE* mRNA levels.

The recognition of the non-canonical poly(A) signals by the 3′-end processing machinery is not completely understood. It is currently believed that auxiliary sequences located either upstream or downstream (DSEs) of the non-canonical poly(A) sites may be able to compensate for a degenerated hexamer. Such sequences may serve to stabilize the poly(A) complex assembly by providing alternative binding for components of the 3′-end processing machinery [50]. A wide scale analysis of human poly(A) signals has in fact shown that many non-canonical poly(A) signals contain upstream A-rich sequences and tend to have a higher frequency of U and GU nucleotides in their DSE compared with canonical poly(A) signals [48]. Knowing that the human *HFE* 3′ UTR has a very high A+U content [25], probably the recognition of its non-canonical poly(A) signals might indeed take advantage of potential A-, U- and/or GU-rich elements.

As indicated above, it is known that mRNAs with multiple poly(A) signals tend to use non-canonical polyadenylation signals (including the common AUUAAA) more often than mRNAs with a single poly(A) hexamer. It has been also shown that the occurrence of non-canonical polyadenylation signals mediates variation in polyadenylation efficiency, thus enabling developmental, physiological and pathological regulation of gene expression [46], [51]–[53]. The occurrence of alternative polyadenylation can also enable gene expression response to physiological stimuli [53], [54]. Recently, it has been clearly shown that alternative polyadenylation is actually a mechanism by which transcripts can lose repressive 3′ UTR elements which is associated to promotion of oncogenic transformation or immune cell activation [55], [56]. These authors have shown that states of increased proliferation are associated with widespread reductions in the 3′ UTRs by alternative polyadenylation, with the shorter mRNA isoforms exhibiting increased stability and typically producing more protein, in part through the loss of microRNA-mediated repression [55], [56]. It was recently shown that the liver-specific microRNA miR-122 clearly affects *HFE* expression, as well as expression of other iron-related genes [57]. In fact, two miR-122 recognition sites were found to be present in the *HFE* 3′ UTR, at about 60 and 260 nts downstream the stop codon, respectively [57]. Nevertheless, these sites are located upstream of the four alternative polyadenylation sites found in the present work and thus they are expected to affect the abundance of the four polyadenylation *HFE* isoforms in a similar fashion.

Our data show that the human *HFE* mRNA expression results from alternative polyadenylation at a GAUAAA non-canonical poly(A) signal at exon six, or at two AUUAAA sites or one AAUAAA site located at exon seven of the *HFE* 3′ UTR. The extended *HFE* 3′ UTR isoforms encompass an intron located more than 55 nts downstream of the native stop codon, which is a feature that induces mRNA destabilization by the NMD mechanism. The present work provides an example of how shortening the 3′ UTR by alternative cleavage and polyadenylation can have the functional consequence of increasing mRNA levels through the loss of the feature that can make this transcript an NMD-target. The hypothesis that alternative polyadenylation and NMD respond to cellular stimuli to coordinately regulate *HFE* mRNA levels in human cells will be the subject of future studies.

## Materials and Methods

### 3′ Rapid amplification of cDNA ends (3′-RACE)

3′-RACE experiments were performed using the BD SMART™ RACE cDNA Amplification Kit (BD Biosciences Clontech) according to the manufacturer's instructions. Briefly, one microgram of total RNA from each tissue was retrotranscribed using 3′-RACE CDS Primer A together with the kit specific components for cDNA synthesis (90 min at 42°C). To cover the entire human *HFE* 3′ UTR, four parallel PCR reactions were performed with a *HFE* specific forward primer (primer #1, #2, #3, or #4; Table 1) and the universal primer of the 3′-RACE amplification kit, as reverse primer. A touchdown PCR program was done as indicated in the user manual. Then, a nested PCR was performed by using primers one forward internal primer (primer #5: EX6F; primer #6: EX6G; primer #7: EX7G; primer #8: EX7H; Fig. 1A and Table 1) and the nested universal primer from the kit. The PCR products from the nested PCR were separated on agarose gels, cloned into the pCR®2.1-TOPO (Invitrogen) and sequenced with BigDye terminator v1.1 sequencing standard kit (Applied Biosystems), using M13 forward and reverse primers and analysed with the ABI Prism 3100 automatic sequencer (Applied Biosystems).

**Table 1:** DNA oligonucleotides used in the current work.

Primer	Sequence (5′→3′)
#1	AGT GAC ACG CAG CCT GCA GAC TCA C
#2	TGG TGC CTT CAT TTG GGA TGC TAC TC
#3	TTC AAC TGT GGT AGC CGA ATT AAT CGT G
#4	GAA TCA CAG GCC ATT GCT GAG CTG CC
#5	TTT CTG AGT TCC TGC ATG CCG GTG ATC C
#6	AGT GAA GTA GGC CGG GCA CGG TGG C
#7	GGC TTC ACT TAC TCT TCT ACC TCA TAA GG
#8	GAT TGA GGA CTG CTG AGA GGT ACA GGC C
#9	TTT TGC GGC CGC ATG GGC CCG CGA GCC AGG CCG
#10	TTT TAT CGA TAG GTC CCA TCC CCA TTG GGC
#11	GAA CAT CAC CAT GAA GTG GCT GAA GG
#12	GGG GTG TTT CTT GAA ATC TCA GCC C
#13	GAA GGG CAG GTG CTT CAG GAT ACC
#14	TTT TTC CGG AAC ATG GTA ACT GTT GCC
#15	GGG CGC TCT TCC GCT TCC TTC CGG ACG CTC ACT GAC GAC TCG
#16	GCG AGT CAG TGA GCG TCC GGA AGG AAG CGG AAG AGC GCC C
#17	CCG AGG GCT ACT GGA AGT AGG GGT ATG ATG GGC AGG
#18	CCT GCC CAT CAT ACC CCT ACT TCC AGT AGC CCT CGG
#19	GGG CTC TAG GGG GTA TCC TCC GGA CCA CGC GCC CTG TAG C
#20	GCT ACA GGG CGC GTG GTC CGG AGG ATA CCC CCT AGA GCC C
#21	CTA CGT CTT AGC TGAACG TGA GTG A
#22	TGT CTC CTT CCC ACA GTG AGT CT
#23	AAG CAT TCT GTC TTG AAG GGC A
#24	CTG AGC TGT ATA TGG TAT CCT GAA GC
#25	CGA GTC CAA GTA CGC CTC ATG
#26	GGT TGT CCT TCA TCT CGT CCA
#27	CCA CTG CTT ACT GGC TTA TCG A
#28	GGG TCT CCC TAT AGT GAG TCG TAT TA
#29	CGA CCA CCA AGC GAA ACA T
#30	GCT TCC ATC CGA GTA CGT GC
#31	GTG GAT CCT GAG AAC TTC AGG CT
#32	CAG CAC ACA GAC CAG CAC GT
#33	CGC AAC CTC CCC TTC TAC G
#34	GGT GAC GGT GAA GCC GAG

### Plasmid constructs

The *HFE* minigenes used in this work comprise all human *HFE* exons and introns 4, 5 and 6. The normal *HFE* minigene (WT) was obtained by sequentially cloning and ligating three distinct human *HFE* fragments. The first fragment, encompassing exon 1 from AUG codon to exon 4, was PCR amplified from *HFE* cDNA by using primers #9 (with a NotI restriction site linker; Table 1) and #10 (with a ClaI restriction site linker; Table 1). An 812 bp fragment was isolated on an agarose gel and cloned into the pCR®2.1-TOPO vector (Invitrogen). Then, this fragment was inserted into pTRE2pur (Clontech) using the NotI and ClaI restriction enzymes. The second fragment of the *HFE* minigene encompasses exon 4 to the 5′-end of exon seven of the human *HFE* gene. It was PCR amplified with the Expand Long Template PCR System (Roche) and primers #11 and #12 (Table 1), using human genomic DNA as template. The corresponding 4455 bp fragment was purified and cloned into pCR®2.1-TOPO vector and subsequently inserted into the pTRE2pur already containing the first *HFE* fragment, by using BstBI (restriction site located in *HFE* exon 4) and EcoRV (restriction site present in *HFE* exon seven) enzymes. The third *HFE* fragment encompasses all exon seven plus a 401 bp downstream fragment. It was PCR amplified by using the Expand Long Template PCR System with primers #13 and #14 (with a Kpn2I restriction site linker; Table 1) and human genomic DNA as template. The resulting 2210 bp fragment was isolated in an agarose gel and purified, cloned into the pCR®2.1-TOPO vector and subsequently subcloned into the pTRE2pur carrying the two previously cloned *HFE* fragments, by using EcoRV and Kpn2I restriction enzymes (the Kpn2I site was previously inserted into the original multicloning site of the pTRE2pur vector at position 1770, by directed mutagenesis using primers #15 and #16; Table 1). All cloned fragments were confirmed through automated sequencing with several human *HFE* specific primers. The NMD-resistant minigene (Del_IVS6) was obtained from the wild-type minigene, by replacing the BsgI and BsmI fragment containing intron 6 with the corresponding BsgI/BsmI cDNA fragment. The nonsense-mutated minigenes (Y138X) carrying the naturally-occurring nonsense mutation TAC→TAG at codon 138 [38] were obtained by site-directed mutagenesis using primers #17 and #18 (Table 1) and the QuickChange® Site-Directed Mutagenesis Kit (Stratagene). All the *HFE* minigenes where finally removed from the pTRE2pur vector and subcloned into the pcDNA3 (Invitrogen) using NotI and Kpn2I restriction enzymes. The Kpn2I site was previously introduced into pcDNA3 vector at position 1310 by site-directed mutagenesis using primers #19 and #20 (Table 1), therefore removing the polyadenylation site of the bovine growth hormone gene present in the vector. The β39 gene was cloned into the pTRE2pur vector (BD Biosciences) as previously described [37].

### Cell culture and transfections

HepG2 cells (DSMZ, ACC 180) were grown in RPMI 1640 medium supplemented with 10% (v/v) fetal bovine serum (FBS). HeLa cells (ATCC, CCL-2) were grown in Dulbecco's modified Eagle's medium (DMEM) supplemented with 10% (v/v) FBS. When appropriated, HeLa and HepG2 cells were treated with cycloheximide (200 µmol/L) and harvested two hours later for RNA isolation.

Transient transfections were performed in HeLa cells using Lipofectamine 2000 Transfection Reagent (Invitrogen), following the manufacturer's instructions, in 35 mm plates, using 1 µg of the test construct DNA and 1 µg of pEGFP vector (BD Biosciences) to control for transfection efficiency. Cells were harvested for RNA and protein lysates, twenty-four hours later.

### Transient transfections of siRNAs

Transfections of HeLa cells with siRNAs were carried out using Lipofectamine 2000 reagent (Invitrogen) according to the manufacturer's instructions, in 60 mm plates, using 200 pmol of siRNA oligonucleotides and 10 µl of transfection reagent. Cells were harvested for RNA and protein extracts at twenty-four, forty-eight and seventy-two hours post-transfection. In the case cells were also transiently transfected with plasmid constructs, siRNAs transfections were performed at twenty-two hours (100 pmol of siRNAs) and concomitantly (50 pmols of siRNAs) with the plasmids transfections. The siRNA oligonucleotides used for transfections [Luciferase (5′-CGUACGCGGAAUACUUCGA-3′) and UPF1 (5′-UUACCGCGUUCUGUGUGAA-3′)] were purchased as annealed, ready-to-use duplexes from MWG. HepG2 cells were transfected with the same siRNAs using 200 pmol of each oligonucleotide and 10 µl of Lipofectamine™ RNAiMAX transfection reagent (Invitrogen), following the reverse-transfection protocol indicated by the manufacturer. Twenty-four, forty-eight and seventy-two hours later, cells were collected for RNA and protein extracts.

### RNA isolation

Total RNA from small intestine, spleen, liver, testis, ovary, duodenum, heart, kidney and peripheral blood mononuclear cells (PBMCs) was purchased (BD Clontech or Ambion). RNA from transfected cells was prepared using the RNeasy mini kit (Qiagen) following the manufacturer's indications. RNA samples were treated with RNase-free DNase I (Ambion) and purified by phenol:chloroform extraction. Before further analyses, mRNA samples isolated from cultured cells transfected with test plasmids were assessed by RT-PCR to reject the hypothesis of activation of cryptic splicing pathway(s) that might affect the human *HFE* mRNA sequence. From all the studied transcripts, a single full-length product was amplified (data not shown), demonstrating a normal splicing pattern.

### Quantitative reverse transcription-coupled real-time PCR (RT-qPCR)

Synthesis of cDNA was carried out using 3 µg of total RNA and SuperScript® III Reverse Transcriptase (Invitrogen), according to the manufacturer's instructions. Real-time PCR was performed in an ABI Prism 7000 Sequence Detection System using SYBR Green Master Mix (Applied Biosystems). Primers were designed using the ABI Primer Express software. Primers were specific for the following transcripts: *HFE* (Exon six: primers #21 and #22; Exon seven: primers #23 and #24; Table 1); G protein pathway suppressor 1 (primers #25 and #26; Table 1); Heterologous 5′ UTR common to all transfected *HFE* minigenes (primers #27 and #28, Table 1); Neomycin resistance gene (primers #29 and #30; Table 1); β-globin gene (primers #31 and #32, Table 1); Puromycin resistance gene (primers #33 and #34, Table 1). The following cycling parameters were used: 10 min at 95°C, and 40 cycles (15 sec at 95°C and 1 min at 65°C) for all transcripts except for β-globin and puromycin resistance transcripts. Quantification of each transcript was performed by the absolute quantification method using serial dilutions of plasmids carrying the corresponding cDNA. These plasmids were generated by introducing PCR fragments into pCR®2.1-TOPO vector. For β-globin and puromycin resistance mRNAs, the parameters used were: 10 min at 95°C, and 40 cycles (15 sec at 95°C and 1 min at 60°C); the quantification was performed using the relative standard curve method (ΔΔ*C*_t_, Applied Biosystems).

### SDS-PAGE and Western blotting

Protein lysates were resolved in 10% SDS-PAGE according to standard protocols and transferred to PVDF membranes (Bio-Rad). Membranes were probed using goat polyclonal anti-UPF1 (Bethyl Labs) at 1∶250 dilution, or mouse monoclonal anti-α-tubulin (as loading control; Sigma). Detection was carried out using secondary peroxidase-conjugated anti-mouse IgG (Bio-Rad) or anti-goat IgG (Sigma) antibodies followed by chemiluminescence assays. For densitometric analysis, films from at least three independent experiments were digitalized and analyzed using ImageJ software.

### Statistical analysis

Results are expressed as mean ± standard deviation. Student's *t* test was used for estimation of statistical significance. Significance for statistical analysis was defined as a *p*<0.05.

## Supporting Information

Figure S1 **Representative agarose gel electrophoreses showing 3′-RACE products from total RNA from different tissues**: duodenum, heart, peripheral blood mononuclear cells (PBMCs), kidney, testis, spleen, small intestine and ovary (A–H, respectively). The 3′-RACE products were obtained by nested PCR using forward primers specified above each lane, the universal primer and the master mix provided by the BD SMART RACE cDNA Amplification Kit (BD Biosciences Clontech). The molecular weight marker (M) is the 1 kb DNA ladder (Invitrogen).
(TIF)

File S1 3′RACE amplicons that do not support *HFE* polyadenylated mRNA and the corresponding sequencing data.
(DOC)
