# Supplementary figures and images for: Alternative Polyadenylation and Nonsense-Mediated Decay Coordinately Regulate the Human HFE mRNA Levels
Source: PLoS One. 2012 Apr 18;7(4):e35461. doi: 10.1371/journal.pone.0035461 (PMC3329446; doi:10.1371/journal.pone.0035461)

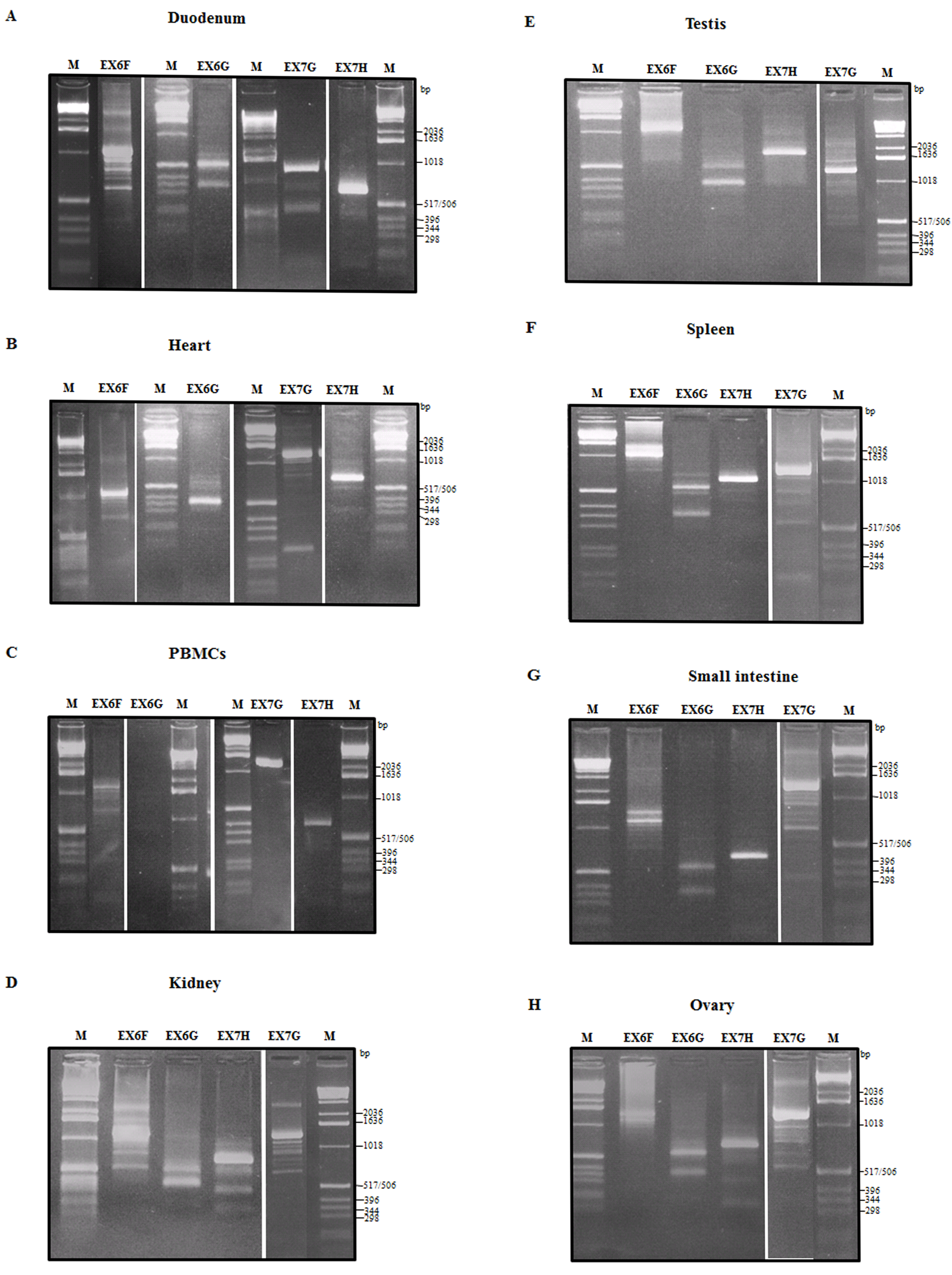

Supplement: Figure S1 — Representative agarose gel electrophoreses showing 3′-RACE products from total RNA from different tissues: duodenum, heart, peripheral blood mononuclear cells (PBMCs), kidney, testis, spleen, small intestine and ovary (A–H, respectively). The 3′-RACE products were obtained by nested PCR using forward primers specified above each lane, the universal primer and the master mix provided by the BD SMART RACE cDNA Amplification Kit (BD Biosciences Clontech). The molecular weight marker (M) is the 1 kb DNA ladder (Invitrogen). (TIF) [file pone.0035461.s001.tif]
