# Supplementary material for: Alternative Polyadenylation and Nonsense-Mediated Decay Coordinately Regulate the Human HFE mRNA Levels
Source: PLoS One. 2012 Apr 18;7(4):e35461. doi: 10.1371/journal.pone.0035461 (PMC3329446; doi:10.1371/journal.pone.0035461)
Supplement: File S1 — 3′RACE amplicons that do not support HFE polyadenylated mRNA and the corresponding sequencing data. (DOC) [file pone.0035461.s002.doc]

**TABLE S1. 3’RACE amplicons that do not support *HFE* polyadenylated mRNA**

| **3’RACE Amplicon** | | | **Tissue** |
| --- | --- | --- | --- |
| **Number** | **3’RACE Primer*** | **Size (bp)** |
| #1 | EX6F | 477 | Kidney |
| #2 | EX6F | >587 | Duodenum, liver, heart, PBMCs, kidney, spleen, small intestine |
| #3 | EX6F | >902 | Duodenum, liver, heart, PBMCs, kidney, spleen, small intestine |
| #4 | EX6F | >1154 | Duodenum, PBMCs, kidney, spleen, small intestine, ovary |
| #5 | EX6G | 345 | Duodenum, liver, heart, kidney, testis, spleen, small intestine, ovary |
| #6 | EX7G | 547 | Duodenum, testis, spleen, small intestine, ovary |
| #7 | EX7G | 560 | Heart, kidney, testis, small intestine, ovary |
| #8 | EX7G | 641 | Heart, kidney, testis, spleen, small intestine, ovary |
| #9 | EX7H | 162 | Duodenum, kidney, ovary |

*3’RACE primer is identified as in Figure 1.

**Sequencing data of the 3’RACE amplicons that do not support polyadenylated *HFE* mRNA:**

**#1.**

CAATCTTGCCnCTTGATTAcCGaGCTCGGAtCCaCTAGTAACGGCCGCCAGTGTGCTGGA

ATTCGCCCTTTTTCTGAGTTCCTGCATGCCGGTGATCCCTAGCTGTGACCTCTCCCCTGG

AACTGTCTCTCATGAACCTCAAGCTGCATCTAGAGGCTTCCTTCATTTCCTCCGTCACCT

CAGAGACATACACCTATGTCATTTCATTTCCTATTTTTGGAAGAGGACTCCTTAAATTTG

GGGGACTTACATGATTCATTTTAACATCTGAGAAAAGCTTTGAACCCTGGGACGTGGCTA

GTCATAACCTTACCAGATTTTTACACATGTATCTATGCATTTTCTGGACCCGTTCAACTT

TTCCTTTGAATCCTCTCTCTGTGTTACCCAGTAACTCATCTGTCACCAAGCCTTGGGGAT

TCTTCCATCTGATTGTGATGTGAGTTGCACAGCTATGAAGGCTGTGCACTGCACGAATGG

AAGAGGCACCTGTCCCAAAAAAAAAAAAAAAAAAAAAAAAAGTACTCTGCGTTGATACCA

CTGCTTAAGGGCGAATTCTGCAGATATCCATCACACTGGCGGCCGCTCGAGCATGCATCT

AGAGGGCCCAATTCGCCCTATAGTGAGTCGTATTACAATTCACTGGCCGTCGTTTTACAA

CGTCGTGACTGGGAAAACCCTGGCGTTACCCAACTTAATCGCCTTGCAGCACATCCCCCT

TTCGCCAGCTGGCGTAATAGCGAAGAGGCCCGCACCGATCGCCCTTCCCAACAGTTGCGC

AGCCTGAATGGCGAATGGACGCGCCCTGTAGCGGCGCATTAAGCGCGGCGGGTGTGGTGG

TTACGCGCAGCGTGACCGCTACACTTGCCAGCGCCCTAGCGCCCGCTCCTTTCGCTTTCT

TCCCTTCCTTTCTCGCCCACGTTCGCCGGCTTTCCCCGTCAAGCTCTAAATCGGGGGCTC

CCTTTAGGGTTTCCGATTTAGTGCTTTACGGCACCCTCGACCCCAAAAACTTGATTAGGG

TGATGGGTTCACGTAGTGGGCCCATCGCCCTGATAGACGGTTTTCGCCCTTTGACGTGAG

TCCACGTTCTTATAGTGGACCTCTGTCCAAACTGGACACACTCACCTATCTCGTCTATTC

TTTTTTGATTAAGGATTGCCGATTCGCCCTATTGTAAAATGAGCCCGTGAAT

Cloned fragment: 477 bp

The human HFE fragment is underlined and the cloning site is grey shaded

Alignment between the cloned sequence (Query) and the human HFE 3’UTR sequence (Subject):

Query 71 TTTCTGAGTTCCTGCATGCCGGTGATCCCTAGCTGTGACCTCTCCCCTGGAACTGTCTCT 130

||||||||||||||||||||||||||||||||||||||||||||||||||||||||||||

Sbjct 8214 TTTCTGAGTTCCTGCATGCCGGTGATCCCTAGCTGTGACCTCTCCCCTGGAACTGTCTCT 8273

Query 131 CATGAACCTCAAGCTGCATCTAGAGGCTTCCTTCATTTCCTCCGTCACCTCAGAGACATA 190

||||||||||||||||||||||||||||||||||||||||||||||||||||||||||||

Sbjct 8274 CATGAACCTCAAGCTGCATCTAGAGGCTTCCTTCATTTCCTCCGTCACCTCAGAGACATA 8333

Query 191 CACCTATGTCATTTCATTTCCTATTTTTGGAAGAGGACTCCTTAAATTTGGGGGACTTAC 250

||||||||||||||||||||||||||||||||||||||||||||||||||||||||||||

Sbjct 8334 CACCTATGTCATTTCATTTCCTATTTTTGGAAGAGGACTCCTTAAATTTGGGGGACTTAC 8393

Query 251 ATGATTCATTTTAACATCTGAGAAAAGCTTTGAACCCTGGGACGTGGCTAGTCATAACCT 310

||||||||||||||||||||||||||||||||||||||||||||||||||||||||||||

Sbjct 8394 ATGATTCATTTTAACATCTGAGAAAAGCTTTGAACCCTGGGACGTGGCTAGTCATAACCT 8453

Query 311 TACCAGATTTTTACACATGTATCTATGCATTTTCTGGACCCGTTCAACTTTTCCTTTGAA 370

||||||||||||||||||||||||||||||||||||||||||||||||||||||||||||

Sbjct 8454 TACCAGATTTTTACACATGTATCTATGCATTTTCTGGACCCGTTCAACTTTTCCTTTGAA 8513

Query 371 TCCTCTCTCTGTGTTACCCAGTAACTCATCTGTCACCAAGCCTTGGGGATTCTTCCATCT 430

||||||||||||||||||||||||||||||||||||||||||||||||||||||||||||

Sbjct 8514 TCCTCTCTCTGTGTTACCCAGTAACTCATCTGTCACCAAGCCTTGGGGATTCTTCCATCT 8573

Query 431 GATTGTGATGTGAGTTGCACAGCTATGAAGGCTGTGCACTGCACGAATGGAAGAGGCACC 490

||||||||||||||||||||||||||||||||||| ||||||||||||||||||||||||

Sbjct 8574 GATTGTGATGTGAGTTGCACAGCTATGAAGGCTGTACACTGCACGAATGGAAGAGGCACC 8633

Query 491 TGTCCCaaaaaaa 503

||||||| |||||

Sbjct 8634 TGTCCCAGAAAAA 8646

**#2.**

GAACGATCCTATAGGGCnATTGGGCCCTCTAGAtGCATGCTCGAGCGGCCGCCAGTGTGA

TGGATATCTGCAGAATTCGCCCTTAAGCAGTGGTATCAACGCAGAGTACTTTTTTTTTTT

TTTTTTTTTTTTTTTTTTCATTTTTATTTTTTTGAGATGGAGTCTCACTCTGTCCCCTAG

GCTGGAGTGCAGTGGCCCAAACTCGGCTCACTGCAAGCTCTGCCTCCTGGGTTCATGCCA

TTCTCCTGCCTCACCCTTCCAAGTAGCTGGGACTACAGATGAGTTACTGGGTAAAACAGA

AAGAGGATTCAAAGGAAAAGTTGAACGGGTCCAAAAAATGCATAGATACATGTGTAAAAA

TCTGGTAAGGTTATGACTAGCCACTCCCCAGGGTTCAAAGTTTTTTTCAGAGGTTAAAAG

GAATCATGTAGGTCCCCCAAATTTAAGGAGTCTTCTTCCCAAAAAGAAAAGAAAATACAC

AGTGTGGTTTCTTTGGTACGGCCAAAAAAAAAAGAACACCCCCCCACCCTCCCCTTTTGT

TAAAAAAAAACTTTCCCGGGGGAAAAAGGCCCCATTCGGGGGCCCCCCCCCCGGGCAAAA

ACAAAAAAAAAAGGGGGTAATTCTCCCCCCCCCCGGGGGCGGGCCTTTTTTTAGGGGACC

CCCGCCCCTGG

Cloned fragment: >587 bp

The HFE fragment is underlined and the cloning site is grey shaded

Alignment between the cloned sequence (Query) and the human HFE 3’UTR sequence (Subject):

Query 275 ACAGATGAGTTACTGGGTAAAACAGAAAGAGGATTCAAAGGAAAAGTTGAACGGGTCCAA 334

|||||||||||||||||||| ||||| ||||||||||||||||||||||||||||||||

Sbjct 8546 ACAGATGAGTTACTGGGTAACACAGAGAGAGGATTCAAAGGAAAAGTTGAACGGGTCCAG 8487

Query 335

AAAATGCATAGATACATGTGTAAAAATCTGGTAAGGTTATGACTAGCCAC-TCCCCAGGG 393

|||||||||||||||||||||||||||||||||||||||||||||||||| |||| ||||

Sbjct 8486

AAAATGCATAGATACATGTGTAAAAATCTGGTAAGGTTATGACTAGCCACGTCCC-AGGG 8428

Query 394 TTCAAAGtttttttCAGAGGTTAAAAGGAATCATGTAGGTCCCCCAAATTTAAGGAGTCT 453

||||||| |||| ||||| ||||||| |||||||||| |||||||||||||||||||||

Sbjct 8427 TTCAAAGCTTTTCTCAGATGTTAAAATGAATCATGTAAGTCCCCCAAATTTAAGGAGTCC 8368

Query 454 TCTTCCCAAAA-AGAAAA-GAAA 474

|||||| |||| || ||| ||||

Sbjct 8367 TCTTCCAAAAATAGGAAATGAAA 8345

Score = 265 bits (143), Expect = 1e-73

Identities = 162/171 (95%), Gaps = 1/171 (1%)

Strand=Plus/Minus

Query 108 ACtttttttttttttttttttttttttttttcatttttatttttttGAGATGGAGTCTCA 167

||||| |||||||||| ||||||| ||||| ||||||||||||||||||||||||||||

Sbjct 9342

ACTTTCTTTTTTTTTTCATTTTTTTATTTTT-ATTTTTATTTTTTTGAGATGGAGTCTCA 9284

Query 168 CTCTGTCCCCTAGGCTGGAGTGCAGTGGCCCAAACTCGGCTCACTGCAAGCTCTGCCTCC 227

||||||| ||||||||||||||||||||| ||||||||||||||||||||||||||||||

Sbjct 9283 CTCTGTCACCTAGGCTGGAGTGCAGTGGCGCAAACTCGGCTCACTGCAAGCTCTGCCTCC 9224

Query 228 TGGGTTCATGCCATTCTCCTGCCTCACCCTTCCAAGTAGCTGGGACTACAG 278

|||||||||||||||||||||||||| |||||| |||||||||||||||||

Sbjct 9223 TGGGTTCATGCCATTCTCCTGCCTCAGCCTTCCGAGTAGCTGGGACTACAG 9173

**#3.**

CAACCCCACCTTGGGTaCCGnCTCGGnCCaCTAGTAACGGCCGCAGTGTGCTGGAATTCG

CCCTTTTTCTGAGTTCCTGCATGCCGGTGATCCCTAGCTGTGACCTCTCCCCTGGAACTG

TCTCTCATGAACCTCAAGCTGCATCTAGAGGCTTCCTTCATTTCCTCCGTCACCTCAGAG

ACATACACCTATGTCATTTCATTTCCTATTTTTGGAAGAGGACTCCTTAAATTTGGGGGA

CTTACATGATTCATTTTAACATCTGAGAAAAGCTTTGAACCCTGGGACGTGGCTAGTCAT

AACCTTACCAGATTTTTACACATGTATCTATGCATTTTCTGGACCCGTTCAACTTTTCCT

TTGAATCCTCTCTCTGTGTTACCCAGTAACTCATCTGTCACCAAGCCTTGGGGATTCTTC

CATCTGATTGTGATGTGAGTTGCACAGCTATGAAGGCTGTACACTGCACGAATGGAAGAG

GCACCTGTCCCAGAAAAAGCATCATGGCTATCTGTGGGTAGTATGATGGGTGTTTTTAGC

AGGTAGGAGGCAAATATCTTGAAAGGGGTTGTGAAGAGGTGTTTTTTCTAATTGGCATGA

AGGTGTCATACAGATTTGCAAAGTTTAATGGTGCCTTCATTTGGGATGCTACTCTAGTAT

TCCAGACCTGAAGAATCACAATAATTTTCTACCTGGTCTCTCCTTGTTCTGATAATGAAA

ATTATGATAAGGATGATAAAAGCACTTACTTCGTGTCCGACTCTTCTGAGCACCTACTTA

CATGCATTACTGCATGCACTTCTTACAATAATTCTATGAGATAGGTACTATTATCCCCAT

TTCTTTTTTAAATGAGGAAAGTGAAGTAGGCCGGGCACGGTGGCTCGCGCCTGTGATCCC

AGCACTTGGAGCCCGAGCGGGTGGATCACCGAGGTTCCAGGGAGAGAATTCCAGAGAAAA

CACCATC

Cloned fragment: >902 bp

The HFE fragment is underlined and the cloning site is grey shaded

Alignment between the cloned sequence (Query) and the human HFE 3’UTR sequence (Subject):

Query 66 TTTCTGAGTTCCTGCATGCCGGTGATCCCTAGCTGTGACCTCTCCCCTGGAACTGTCTCT 125

||||||||||||||||||||||||||||||||||||||||||||||||||||||||||||

Sbjct 8214 TTTCTGAGTTCCTGCATGCCGGTGATCCCTAGCTGTGACCTCTCCCCTGGAACTGTCTCT 8273

Query 126 CATGAACCTCAAGCTGCATCTAGAGGCTTCCTTCATTTCCTCCGTCACCTCAGAGACATA 185

||||||||||||||||||||||||||||||||||||||||||||||||||||||||||||

Sbjct 8274 CATGAACCTCAAGCTGCATCTAGAGGCTTCCTTCATTTCCTCCGTCACCTCAGAGACATA 8333

Query 186 CACCTATGTCATTTCATTTCCTATTTTTGGAAGAGGACTCCTTAAATTTGGGGGACTTAC 245

||||||||||||||||||||||||||||||||||||||||||||||||||||||||||||

Sbjct 8334 CACCTATGTCATTTCATTTCCTATTTTTGGAAGAGGACTCCTTAAATTTGGGGGACTTAC 8393

Query 246 ATGATTCATTTTAACATCTGAGAAAAGCTTTGAACCCTGGGACGTGGCTAGTCATAACCT 305

||||||||||||||||||||||||||||||||||||||||||||||||||||||||||||

Sbjct 8394 ATGATTCATTTTAACATCTGAGAAAAGCTTTGAACCCTGGGACGTGGCTAGTCATAACCT 8453

Query 306 TACCAGATTTTTACACATGTATCTATGCATTTTCTGGACCCGTTCAACTTTTCCTTTGAA 365

||||||||||||||||||||||||||||||||||||||||||||||||||||||||||||

Sbjct 8454 TACCAGATTTTTACACATGTATCTATGCATTTTCTGGACCCGTTCAACTTTTCCTTTGAA 8513

Query 366 TCCTCTCTCTGTGTTACCCAGTAACTCATCTGTCACCAAGCCTTGGGGATTCTTCCATCT 425

||||||||||||||||||||||||||||||||||||||||||||||||||||||||||||

Sbjct 8514 TCCTCTCTCTGTGTTACCCAGTAACTCATCTGTCACCAAGCCTTGGGGATTCTTCCATCT 8573

Query 426 GATTGTGATGTGAGTTGCACAGCTATGAAGGCTGTACACTGCACGAATGGAAGAGGCACC 485

||||||||||||||||||||||||||||||||||||||||||||||||||||||||||||

Sbjct 8574 GATTGTGATGTGAGTTGCACAGCTATGAAGGCTGTACACTGCACGAATGGAAGAGGCACC 8633

Query 486 TGTCCCAGAAAAAGCATCATGGCTATCTGTGGGTAGTATGATGGGTGTTTTTAGCAGGTA 545

||||||||||||||||||||||||||||||||||||||||||||||||||||||||||||

Sbjct 8634 TGTCCCAGAAAAAGCATCATGGCTATCTGTGGGTAGTATGATGGGTGTTTTTAGCAGGTA 8693

Query 546 GGAGGCAAATATCTTGAAAGGGGTTGTGAAGAGGTGTTTTTTCTAATTGGCATGAAGGTG 605

||||||||||||||||||||||||||||||||||||||||||||||||||||||||||||

Sbjct 8694 GGAGGCAAATATCTTGAAAGGGGTTGTGAAGAGGTGTTTTTTCTAATTGGCATGAAGGTG 8753

Query 606 TCATACAGATTTGCAAAGTTTAATGGTGCCTTCATTTGGGATGCTACTCTAGTATTCCAG 665

||||||||||||||||||||||||||||||||||||||||||||||||||||||||||||

Sbjct 8754 TCATACAGATTTGCAAAGTTTAATGGTGCCTTCATTTGGGATGCTACTCTAGTATTCCAG 8813

Query 666 ACCTGAAGAATCACAATAATTTTCTACCTGGTCTCTCCTTGTTCTGATAATGAAAATTAT 725

||||||||||||||||||||||||||||||||||||||||||||||||||||||||||||

Sbjct 8814 ACCTGAAGAATCACAATAATTTTCTACCTGGTCTCTCCTTGTTCTGATAATGAAAATTAT 8873

Query 726 GATAAGGATGATAAAAGCACTTACTTCGTGTCCGACTCTTCTGAGCACCTACTTACATGC 785

||||||||||||||||||||||||||||||||||||||||||||||||||||||||||||

Sbjct 8874 GATAAGGATGATAAAAGCACTTACTTCGTGTCCGACTCTTCTGAGCACCTACTTACATGC 8933

Query 786 ATTACTGCATGCACTTCTTACAATAATTCTATGAGATAGGTACTATTATCCCCATTTCTT 845

||||||||||||||||||||||||||||||||||||||||||||||||||||||||||||

Sbjct 8934 ATTACTGCATGCACTTCTTACAATAATTCTATGAGATAGGTACTATTATCCCCATTTCTT 8993

Query 846 TTTTAAATGAGGAAAGTGAAGTAGGCCGGGCACGGTGGCTCGCGCCTGTGATCCCAGCAC 905

|||||||||| |||||||||||||||||||||||||||||| ||||||| ||||||||||

Sbjct 8994 TTTTAAATGAAGAAAGTGAAGTAGGCCGGGCACGGTGGCTCACGCCTGTAATCCCAGCAC 9053

Query 906 TT-GG-AGCCCGA-GCGGGTGGATCACCGAGGT 935

|| || || || | ||||||||||||| |||||

Sbjct 9054 TTTGGGAGGCCAAAGCGGGTGGATCAC-GAGGT 9085

**#4.**

CATCAACCCAACCTTGGTaCCGaGCTCGGnCCaCTAGTAACGGCCGCCAGTGTGCTGGAA

TTCGCCCTTAAGCAGTGGTATCAACGCAGAGTACTTTTTTTTTTTTTTTTTTTTTCTACA

TAACTTTTCTAACCACAAAAAAAGAAAATGGTTTAAAAGAAnAGATGAGATATCTTTGCT

AAAATTTAATGCCTAAAGAAnAAACTTCTGAGCTGTATATGGTATCCTGAAGCACCTGCC

CTTCAAGACAGAATGCTTGTACCACATTTATGCAGCCAAGTGCATGTAGTAACATAAAGT

AAACACATGCCATCTGGATATATATATTAAGACTCTTTTGACGGCTGGGCAGGGTGGCTC

AAAAGAGTCGGACACGAAGTAAGTGCTTTTATCATCCTTATCATAATTTTCATTATCAGA

ACAAGGAGAGACCAGGTAGAAAATTATTGTGATTCTTCAGGTCTGGAATACTAGAGTAGC

ATCCCAAATGAAGGCACCATTAAACTTTGCAAATCTGTATGACACCTTCATGCCAATTAG

AAAAAACACCTCTTCACAACCCCTTTCAAGATATTTGCCTCCTACCTGCTAAAAACACCC

ATCATACTACCCACAGATAGCCATGATGCTTTTTCTGGGACAGGTGCCTCTTCCATTCGT

GCAGTGTACAGCCTTCATAGCTGTGCAACTCACATCACAATCAGATGGAAGAATCCCCAA

GGCTTGGTGACAGATGAGTTACTGGGTAACACAGAGAGAGGATTCAAAGGAAAAGTTGAA

CGGGTCCAGAAAATGCATAGATACATGTGTAAAAATCTGGTAAGGTTATGACTAGCCACG

TCCCAGGGTTCAAAGCTTTTCTCAGATGTTAAAATGAATCATGTAAGTCCCCCAAATTTA

AGGGAGTCCTCTTCCAAAAATAGGAAATGAAATGACATAGGTGTATGTCTTCTGAGGTGA

CGGAGGAAATGAAGGAAGCCCTCTAGATGCAGCTTGGAGGTTTCATGAGAGACAGTTCCA

GGGGAGAGGTCAACAGCTAGGGGATTCACCCGCCATGCCGGACCTCAGAAAAAGGGCGGA

TTCTTGCAAGATAATCCATCCACCACTGGCCGGCCGCCTCGAGCATGGCATCTAGAGGCC

CATTTCCGCCCCTAATACGTGAGTCCGNNTACATNACTTGNCGCTTCGTTTACCAACGTC

CGTAGACCTGGNAAAACACTCCN

Cloned fragment: >1154 bp

The HFE fragment is underlined and the cloning site is grey shaded

Alignment between the cloned sequence (Query) and the human HFE 3’UTR sequence (Subject):

Query 353 GGTGGCTCAAAAGAGTCGGACACGAAGTAAGTGCTTTTATCATCCTTATCATAATTTTCA 412

||| ||||| ||||||||||||||||||||||||||||||||||||||||||||||||||

Sbjct 8922

GGT-GCTCAGAAGAGTCGGACACGAAGTAAGTGCTTTTATCATCCTTATCATAATTTTCA 8864

Query 413 TTATCAGAACAAGGAGAGACCAGGTAGAAAATTATTGTGATTCTTCAGGTCTGGAATACT 472

||||||||||||||||||||||||||||||||||||||||||||||||||||||||||||

Sbjct 8863 TTATCAGAACAAGGAGAGACCAGGTAGAAAATTATTGTGATTCTTCAGGTCTGGAATACT 8804

Query 473 AGAGTAGCATCCCAAATGAAGGCACCATTAAACTTTGCAAATCTGTATGACACCTTCATG 532

||||||||||||||||||||||||||||||||||||||||||||||||||||||||||||

Sbjct 8803 AGAGTAGCATCCCAAATGAAGGCACCATTAAACTTTGCAAATCTGTATGACACCTTCATG 8744

Query 533 CCAATTAGAAAAAACACCTCTTCACAACCCCTTTCAAGATATTTGCCTCCTACCTGCTAA 592

||||||||||||||||||||||||||||||||||||||||||||||||||||||||||||

Sbjct 8743 CCAATTAGAAAAAACACCTCTTCACAACCCCTTTCAAGATATTTGCCTCCTACCTGCTAA 8684

Query 593 AAACACCCATCATACTACCCACAGATAGCCATGATGCTTTTTCTGGGACAGGTGCCTCTT 652

||||||||||||||||||||||||||||||||||||||||||||||||||||||||||||

Sbjct 8683 AAACACCCATCATACTACCCACAGATAGCCATGATGCTTTTTCTGGGACAGGTGCCTCTT 8624

Query 653 CCATTCGTGCAGTGTACAGCCTTCATAGCTGTGCAACTCACATCACAATCAGATGGAAGA 712

||||||||||||||||||||||||||||||||||||||||||||||||||||||||||||

Sbjct 8623 CCATTCGTGCAGTGTACAGCCTTCATAGCTGTGCAACTCACATCACAATCAGATGGAAGA 8564

Query 713 ATCCCCAAGGCTTGGTGACAGATGAGTTACTGGGTAACACAGAGAGAGGATTCAAAGGAA 772

||||||||||||||||||||||||||||||||||||||||||||||||||||||||||||

Sbjct 8563 ATCCCCAAGGCTTGGTGACAGATGAGTTACTGGGTAACACAGAGAGAGGATTCAAAGGAA 8504

Query 773 AAGTTGAACGGGTCCAGAAAATGCATAGATACATGTGTAAAAATCTGGTAAGGTTATGAC 832

||||||||||||||||||||||||||||||||||||||||||||||||||||||||||||

Sbjct 8503 AAGTTGAACGGGTCCAGAAAATGCATAGATACATGTGTAAAAATCTGGTAAGGTTATGAC 8444

Query 833 TAGCCACGTCCCAGGGTTCAAAGCTTTTCTCAGATGTTAAAATGAATCATGTAAGTCCCC 892

||||||||||||||||||||||||||||||||||||||||||||||||||||||||||||

Sbjct 8443 TAGCCACGTCCCAGGGTTCAAAGCTTTTCTCAGATGTTAAAATGAATCATGTAAGTCCCC 8384

Query 893 CAAATTTAAGGGAGTCCTCTTCCAAAAATAGGAAATGAAATGACATAGGTGTATGTCTTC 952

||||||||||| |||||||||||||||||||||||||||||||||||||||||||||| |

Sbjct 8383

CAAATTTAAGG-AGTCCTCTTCCAAAAATAGGAAATGAAATGACATAGGTGTATGTCT-C 8326

Query 953 TGAGGTGACGGAGGAAATGAAGGAAGCCCTCTAGATGCAGCTTGGAGGTTTCATGAGAGA 1012

|||||||||||||||||||||||||||| ||||||||||||||| ||||| |||||||||

Sbjct 8325

TGAGGTGACGGAGGAAATGAAGGAAGCC-TCTAGATGCAGCTTG-AGGTT-CATGAGAGA 8269

Query 1013 CAGTTCCAGGGGAGAGGTCAACAGCTAGGGGATTCACCCGCCATGCCGGACCTCAGAAA 1071

|||||||||||||||||||| ||||||||| || |||| || |||| ||| ||||||||

Sbjct 8268

CAGTTCCAGGGGAGAGGTCA-CAGCTAGGG-AT-CACCGGC-ATGCAGGAACTCAGAAA 8214

Score = 459 bits (248), Expect = 9e-132

Identities = 250/252 (99%), Gaps = 0/252 (0%)

Strand=Plus/Minus

Query 110 ttttttCTACATAACTTTTCTAACCACaaaaaaagaaaatggtttaaaagaanagatgag 169

|||||||||||||||||||||||||||||||||||||||||||||||||||| |||||||

Sbjct 10475 TTTTTTCTACATAACTTTTCTAACCACAAAAAAAGAAAATGGTTTAAAAGAAGAGATGAG 10416

Query 170 atatctttgctaaaatttaatgcctaaagaanaaaCTTCTGAGCTGTATATGGTATCCTG 229

||||||||||||||||||||||||||||||| ||||||||||||||||||||||||||||

Sbjct 10415 ATATCTTTGCTAAAATTTAATGCCTAAAGAAGAAACTTCTGAGCTGTATATGGTATCCTG 10356

Query 230 AAGCACCTGCCCTTCAAGACAGAATGCTTGTACCACATTTATGCAGCCAAGTGCATGTAG 289

||||||||||||||||||||||||||||||||||||||||||||||||||||||||||||

Sbjct 10355 AAGCACCTGCCCTTCAAGACAGAATGCTTGTACCACATTTATGCAGCCAAGTGCATGTAG 10296

Query 290 TAACATAAAGTAAACACATGCCATCTGGATATATATATTAAGACTCTTTTGACGGCTGGG 349

||||||||||||||||||||||||||||||||||||||||||||||||||||||||||||

Sbjct 10295 TAACATAAAGTAAACACATGCCATCTGGATATATATATTAAGACTCTTTTGACGGCTGGG 10236

Query 350 CAGGGTGGCTCA 361

||||||||||||

Sbjct 10235 CAGGGTGGCTCA 10224

**#5.**

GACGACTCCCTATAGGGCGAATTGGGCCCTCTAGATGCAAGCTCGAGCGGCCGCCAGTGT

GATGGATATCTGCAGAATTCGCCCTTAAGCAGTGGTATCAACGCAGAGTACTTTTTTTTT

TTTTTTTTTTTTTTTTTTTTGAGATGGAGTCTCACTCTGTCACCTAGGCTGGAGTGCAGT

GGCGCAAACTCGGCTCACTGCAAGCTCTGCCTCCTGGGTTCATGCCATTCTCCTGCCTCA

GCCTTCCGAGTAGCTGGGACCACAGGCGTCTGCCACCACGCCCAGCTAATTTTTTGTATT

TTTATTAGAGATGGGGTTTCACCATGTTAGCCAGGATGGTCTCGATCTCCTGACCTCGTG

ATCCACCCGCTTTGGCCTCCCAAAGTGCTGGGATTACAGGCGTGAGCCACCGTGCCCGGC

CTACTTCACTAAGGGCGAATTCCAGCACACTGGCGGCCGTTACTAGTGGATCCGAGCTCG

GTACCAAGCTTGGCGTAATCATGGTCATAGCTGTTTCCTGTGTGAAATTGTTATCCGCTC

ACAATTCCACACAACATACGAGCCGGAAGCATAAAGTGTAAAGCCTGGGGTGCCTAATGA

GTGAGCTAACTCACATTAATTGCGTTGCGCTCACTGCCCGCTTTCCAGTCGGGAAACCTG

TCGTGCCAGCTGCATTAATGAATCGGCCAACGCGCGGGGAGAGGCGGTTTGCGTATTGGG

CGCTCTTCCGCTTCCTCGCTCACTGACTCGCTGCGCTCGGTCGTTCGGCTGCGGCGAGCG

GTATCAGCTCACTCAAAGGCGGTAATACGGTTATCCACAGAATCAGGGGATAACGCAGGA

AAGAACATGTGAGCAAAAGGCCAGCAAAAGGCCAGGAACCGTAAAAAGGCCGCGTTGCTG

GCGTTTTTCCATAGGCTTCCGCCCCCCCCTGACGAGCATCACAAAAATCGACGCTCAAGT

CAGAGTGGCGAAACCCCGACAGGACTATAAAGATACCAGGCGTTTCCCCCTGGAAGCCTC

CCTCGTGCGCTCTCCTGTTCCGACCTGCCGCTTTACCGGAATACCTGTCCGCCTTTCTCC

CTTCGGGAAGCGGTGGCGCTTTCCTTAGCTAGCCTGTAGTATCCAGTCGTGTAGTCGTTT

CGCTCAGCTGGCTGGTGACGAATCCCGGTCGCTCGAACCGCTGGCCT

Cloned fragment: 345 bp

The human HFE fragment is underlined and the cloning site is grey shaded

Alignment between the cloned sequence (Query) and the human HFE 3’UTR sequence (Subject):

Query 114 tttttttttttttttttttttttttttGAGATGGAGTCTCACTCTGTCACCTAGGCTGGA 173

||||||| ||||| ||||| ||||||||||||||||||||||||||||||||||||||||

Sbjct 9324 TTTTTTTATTTTTATTTTTATTTTTTTGAGATGGAGTCTCACTCTGTCACCTAGGCTGGA 9265

Query 174 GTGCAGTGGCGCAAACTCGGCTCACTGCAAGCTCTGCCTCCTGGGTTCATGCCATTCTCC 233

||||||||||||||||||||||||||||||||||||||||||||||||||||||||||||

Sbjct 9264 GTGCAGTGGCGCAAACTCGGCTCACTGCAAGCTCTGCCTCCTGGGTTCATGCCATTCTCC 9205

Query 234 TGCCTCAGCCTTCCGAGTAGCTGGGACCACAGGCGTCTGCCACCACGCCCAGCTAATTTT 293

||||||||||||||||||||||||||| ||||||||||||||||||||||||||||||||

Sbjct 9204 TGCCTCAGCCTTCCGAGTAGCTGGGACTACAGGCGTCTGCCACCACGCCCAGCTAATTTT 9145

Query 294 TTGTATTTTTATTAGAGATGGGGTTTCACCATGTTAGCCAGGATGGTCTCGATCTCCTGA 353

||||||||||||||||||||||||||||||||||||||||||||||||||||||||||||

Sbjct 9144 TTGTATTTTTATTAGAGATGGGGTTTCACCATGTTAGCCAGGATGGTCTCGATCTCCTGA 9085

Query 354 CCTCGTGATCCACCCGCTTTGGCCTCCCAAAGTGCTGGGATTACAGGCGTGAGCCACCGT 413

||||||||||||||||||||||||||||||||||||||||||||||||||||||||||||

Sbjct 9084 CCTCGTGATCCACCCGCTTTGGCCTCCCAAAGTGCTGGGATTACAGGCGTGAGCCACCGT 9025

Query 414 GCCCGGCCTACTTCACT 430

|||||||||||||||||

Sbjct 9024 GCCCGGCCTACTTCACT 9008

**#6.**

ACACCCCAGCCTTGGTACcGaGCTCGGnCCaCTAGTAACGGCCGCCAGTGTGCTGGAATT

CGCCCTTGGCTTCACTTACTCTTCTACCTCATAAGGAATATGTTACAATTAATTTATTAG

GTAAGCATTTGTTTTATATTGGTTTTATTTCACCTGGGCTGAGATTTCAAGAAACACCCC

AGTCTTCACAGTAACACATTTCACTAACACATTTACTAAACATCAGCAACTGTGGCCTGT

TAATTTTTTTAATAGAAATTTTAAGTCCTCATTTTCTTTCGGTGTTTTTTAAGCTTAATT

TTTCTGGCTTTATTCATAAATTCTTAAGGTCAACTACATTTGAAAAATCAAAGACCTGCA

TTTTAAATTCTTATTCACCTCTGGCAAAACCATTCACAAACCATGGTAGTAAAGAGAAGG

GTGACACCTGGTGGCCATAGGTAAATGTACCACGGTGGTCCGGTGACCAGAGATGCAGCG

CTGAGGGTTTTCCTGAAGGTAAAGGAATAAAGAATGGGTGGAGGGGCGTGCACTGGAAAT

CACTTGTAGAGAAAAGCCCCAAAAAAAAAAAAAAAAAAAAAAAAAAAAGTACTCTGCGTT

GATACCACTGCTTAAGGGCGAATTCTGCAGATATCCATCACACTGGCGGCCGCTCGAGCA

TGCATCTAGAGGGCCCAATTCGCCCTATAGTGAGTCGTATTACAATTCACTGGCCGTCGT

TTTACAACGTCGTGACTGGGAAAACCCTGGCGTTACCCAACTTAATCGCCTTGCAGCACA

TCCCCCTTTCGCCAGCTGGCGTAATAGCGAAGAGGCCCGCACCGATCGCCCTTCCCAACA

GTTGCGCAGCCTGAATGGCGAATGGACGCGCCCTGTAGCGGCGCATTAAGCGCGGCGGGT

GTGGTGGTTACGCGCAGCGTGACCGCTACACTTGCCAGCGCCCTAGCGCCCGCTCCTTTC

GCTTTCTTCCCTTCCTTTCTCGGCCACGTTCGCCGGCTTTCCCCGTCAAGCTCTAAATCG

GGGGCTCCCTTTAGGGTTCGGATTTAGTGCTTTACGGGCACTCGACCCCAAAAACTGGAT

TAGGGTGATGGATCACGTATGGACATCGCCCTGATAGACGGTTTTCGCCCTTGGACGGTG

GGAGTCCAGTCCTTCATATGGAACTCTTGTTCAACTGGAAACAACACT

Cloned fragment: 547 bp

The human HFE fragment is underlined and the cloning site is grey shaded

Alignment between the cloned sequence (Query) and the human HFE 3’UTR sequence (Subject):

Query 68 GGCTTCACTTACTCTTCTACCTCATAAGGAATATGTTACAATTAATTTATTAGGTAAGCA 127

||||||||||||||||||||||||||||||||||||||||||||||||||||||||||||

Sbjct 10918 GGCTTCACTTACTCTTCTACCTCATAAGGAATATGTTACAATTAATTTATTAGGTAAGCA 10977

Query 128 TTTGTTTTATATTGGTTTTATTTCACCTGGGCTGAGATTTCAAGAAACACCCCAGTCTTC 187

||||||||||||||||||||||||||||||||||||||||||||||||||||||||||||

Sbjct 10978 TTTGTTTTATATTGGTTTTATTTCACCTGGGCTGAGATTTCAAGAAACACCCCAGTCTTC 11037

Query 188 ACAGTAACACATTTCACTAACACATTTACTAAACATCAGCAACTGTGGCCTGttaatttt 247

||||||||||||||||||||||||||||||||||||||||||||||||||||||||||||

Sbjct 11038 ACAGTAACACATTTCACTAACACATTTACTAAACATCAGCAACTGTGGCCTGTTAATTTT 11097

Query 248 tttaatagaaattttaagtcctcattttctttcggtgttttttaagcttaatttttCTGG 307

||||||||||||||||||||||||||||||||||||||||||||||||||||||||||||

Sbjct 11098 TTTAATAGAAATTTTAAGTCCTCATTTTCTTTCGGTGTTTTTTAAGCTTAATTTTTCTGG 11157

Query 308 CTTTATTCATAAATTCTTAAGGTCAACTACATTTGAAAAATCAAAGACCTGCATTTTAAA 367

||||||||||||||||||||||||||||||||||||||||||||||||||||||||||||

Sbjct 11158 CTTTATTCATAAATTCTTAAGGTCAACTACATTTGAAAAATCAAAGACCTGCATTTTAAA 11217

Query 368 TTCTTATTCACCTCTGGCAAAACCATTCACAAACCATGGTAGTAAAGAGAAGGGTGACAC 427

||||||||||||||||||||||||||||||||||||||||||||||||||||||||||||

Sbjct 11218 TTCTTATTCACCTCTGGCAAAACCATTCACAAACCATGGTAGTAAAGAGAAGGGTGACAC 11277

Query 428 CTGGTGGCCATAGGTAAATGTACCACGGTGGTCCGGTGACCAGAGATGCAGCGCTGAGGG 487

||||||||||||||||||||||||||||||||||||||||||||||||||||||||||||

Sbjct 11278 CTGGTGGCCATAGGTAAATGTACCACGGTGGTCCGGTGACCAGAGATGCAGCGCTGAGGG 11337

Query 488 TTTTCCTGAAGGTAAAGGAATAAAGAATGGGTGGAGGGGCGTGCACTGGAAATCACTTGT 547

||||||||||||||||||||||||||||||||||||||||||||||||||||||||||||

Sbjct 11338 TTTTCCTGAAGGTAAAGGAATAAAGAATGGGTGGAGGGGCGTGCACTGGAAATCACTTGT 11397

Query 548 AGAGAAAAGCCCC 560

|||||||||||||

Sbjct 11398 AGAGAAAAGCCCC 11410

**#7.**

TCTACGCCGACCTTGTTACCGAGCTCGGnCCaCTAGTAACGGCCGGCAGTGTGCTGGAAT

TCGGCTTCACTTACTCTTCTACCTcATAAGGAATATGTTACAATTAATTTATTAGGTAAG

CATTTGTTTTATATTGGTTTTATTTCACCTGGGCTGAGATTTCAAGAAACACCCCAGTCT

TCACAGTAACACATTTCACTAACACATTTACTAAACATCAGCAACTGTGGCCTGTTAATT

TTTTTAATAGAAATTTTAAGTCCTCATTTTCTTTCGGTGTTTTTTAAGCTTAATTTTTCT

GGCTTTATTCATAAATTCTTAAGGTCAACTACATTTGAAAAATCAAAGACCTGCATTTTA

AATTCTTATTCACCTCTGGCAAAACCATTCACAAACCATGGTAGTAAAGAGAAGGGTGAC

ACCTGGTGGCCATAGGTAAATGTACCACGGTGGTCCGGTGACCAGAGATGCAGCGCTGAG

GGTTTTCCTGAAGGTAAAGGAATAAAGAATGGGTGGAGGGGCGTGCACTGGAAATCGCTT

GTAGAGAAAAGCCCCTGAAAATTTGAGAAAAAAAAAAAAAAAAAAAAAAAAAAAAAGTAC

TCTGCGTTGATACCACTGCTTAAGCCGAATTCTGCAGATATCCATCACACTGGCGGCCGC

TCGAGCATGCATCTAGAGGGCCCAATTCGCCCTATAGTGAGTCGTATTACAATTCACTGG

CCGTCGTTTTACAACGTCGTGACTGGGAAAACCCTGGCGTTACCCAACTTAATCGCCTTG

CAGCACATCCCCCTTTCGCCAGCTGGCGTAATAGCGAAGAGGCCCGCACCGATCGCCCTT

CCCAACAGTTGCGCAGCCTGAATGGCGAATGGACGCGCCCTGTAGCGGCGCATTAAGCGC

GGCGGGTTGTGGTGGTTACGCGCAGCGTGACCGCTACACTTGCCAGCGCCCTAGCGCCCG

CTCCTTTCGCTTTTCTTCCCTTCCTTTTCTCGCCACGTTCGCCGGCTTTCCCCGTCAAGC

TCTAATCCGGGGGCCTCCCTTTAGGGTTCCCGATTAGTTGCTTTACCGGCACCCTCGACC

CCAAAACCTTGATTAGGGTGATGGTTTCACGTATTGGGCATTCGCTTGATAAGAACCGTT

TTCGCCTGACGTGGAGTCCACGTTCCTTTATAGTGACACCCCTTGT

Cloned fragment: 560 bp

The human HFE fragment is underlined and the cloning site is grey shaded

Alignment between the cloned sequence (Query) and the human HFE 3’UTR sequence (Subject):

Query 63 GGCTTCACTTACTCTTCTACCTCATAAGGAATATGTTACAATTAATTTATTAGGTAAGCA 122

||||||||||||||||||||||||||||||||||||||||||||||||||||||||||||

Sbjct 10918 GGCTTCACTTACTCTTCTACCTCATAAGGAATATGTTACAATTAATTTATTAGGTAAGCA 10977

Query 123 TTTGTTTTATATTGGTTTTATTTCACCTGGGCTGAGATTTCAAGAAACACCCCAGTCTTC 182

||||||||||||||||||||||||||||||||||||||||||||||||||||||||||||

Sbjct 10978 TTTGTTTTATATTGGTTTTATTTCACCTGGGCTGAGATTTCAAGAAACACCCCAGTCTTC 11037

Query 183 ACAGTAACACATTTCACTAACACATTTACTAAACATCAGCAACTGTGGCCTGttaatttt 242

||||||||||||||||||||||||||||||||||||||||||||||||||||||||||||

Sbjct 11038 ACAGTAACACATTTCACTAACACATTTACTAAACATCAGCAACTGTGGCCTGTTAATTTT 11097

Query 243 tttaatagaaattttaagtcctcattttctttcggtgttttttaagcttaatttttCTGG 302

||||||||||||||||||||||||||||||||||||||||||||||||||||||||||||

Sbjct 11098 TTTAATAGAAATTTTAAGTCCTCATTTTCTTTCGGTGTTTTTTAAGCTTAATTTTTCTGG 11157

Query 303 CTTTATTCATAAATTCTTAAGGTCAACTACATTTGAAAAATCAAAGACCTGCATTTTAAA 362

||||||||||||||||||||||||||||||||||||||||||||||||||||||||||||

Sbjct 11158 CTTTATTCATAAATTCTTAAGGTCAACTACATTTGAAAAATCAAAGACCTGCATTTTAAA 11217

Query 363 TTCTTATTCACCTCTGGCAAAACCATTCACAAACCATGGTAGTAAAGAGAAGGGTGACAC 422

||||||||||||||||||||||||||||||||||||||||||||||||||||||||||||

Sbjct 11218 TTCTTATTCACCTCTGGCAAAACCATTCACAAACCATGGTAGTAAAGAGAAGGGTGACAC 11277

Query 423 CTGGTGGCCATAGGTAAATGTACCACGGTGGTCCGGTGACCAGAGATGCAGCGCTGAGGG 482

||||||||||||||||||||||||||||||||||||||||||||||||||||||||||||

Sbjct 11278 CTGGTGGCCATAGGTAAATGTACCACGGTGGTCCGGTGACCAGAGATGCAGCGCTGAGGG 11337

Query 483 TTTTCCTGAAGGTAAAGGAATAAAGAATGGGTGGAGGGGCGTGCACTGGAAATCGCTTGT 542

|||||||||||||||||||||||||||||||||||||||||||||||||||||| |||||

Sbjct 11338 TTTTCCTGAAGGTAAAGGAATAAAGAATGGGTGGAGGGGCGTGCACTGGAAATCACTTGT 11397

Query 543 AGAGAAAAGCCCCTGAAAATTTGAGaaaaaaaaaaaaaaa 582

||||||||||||||||||||||||||||| ||| || |||

Sbjct 11398 AGAGAAAAGCCCCTGAAAATTTGAGAAAACAAACAAGAAA 11437

**#8.**

ACCCCCGACCTTGGTaCCGAgCCTCGGnCCaCTAGTAACGGCTCGCCAGTGTGCTGGAAT

TCGCCCTTGGCTTCaCTTACTCTTCTACCTCATAAGGAATATGTTACAATTAATTCATTA

GGTAAGCATTTGTTTTATATTGGTTTTATTTCACCTGGGCTGAGATTTCAAGAAACACCC

AGTCTTCACAGTAACACATTTCACTAACACATTTACTAAACATCAGCAACTGTGGCCTGT

TAATTTTTTTAATAGAAATTTTAAGTCCTCATTTTCTTTCGGTGTTTTTTAAGCTTAATT

TTTCTGGCTTTATTCATAAATTCTTAAGGTCGACTACATTTGAAAAATCAAAGACCTGCA

TTTTAAATTCTTATTCACCTCTGGCAAAACCATTCACAAACCATGGTAGTAAAGAGAAGG

GTGACACCTGGTGGCCATAGGTAAATGTACCACGGTGGTCCGGTGACCAGAGATGCAGCG

CTGAGGGTTTTCCTGAAGGTAAAGGAATAAAGAATGGGTGGAGGGGCGTGCACTGGAAAT

CACTTGTAGAGAAAAGCCCCTGAAAATTTGAGAAAACAAACAAGAAACTACTTACCAGCT

ATTTGAATTGCTGGAATCACAGGCCATTGCTGAGCTGCCTGAACTGGGAACACAACAGAA

AAAAAAAAAAAAAAAAAAAAAAAGTACTCTGCGTTGATACCACTGCTTAAGGGCGAATTC

TGCAGATATCCATCACACTGGCGGCCGCTCGAGCATGCATCTAGAGGGCCCAATTCGCCC

TATAGTGAGTCGTATTACAATTCACTGGCCGTCGTTTTACAACGTCGTGACTGGGAAAAC

CCTGCGTTACCCAACTTAATCGCCTTGCAGCACATCCCCCCTTTCGCCAGCTGGCGTAAT

AGCGAAGAGGCCCCGCACCGATCGCCCTTCCCAACAGTTGCGCAGCCCTGAATGGCGAAT

GACGCGCCTGTAGCGGCGCATTAAAGCGCGGCGGGTTGTTGGTGGTTACGCGCAGCGGTG

ACCGCTAACACTTTG

Cloned fragment: 641 bp

The human HFE fragment is underlined and the cloning site is grey shaded

Alignment between the cloned sequence (Query) and the human HFE 3’UTR sequence (Subject):

Query 69 GGCTTCACTTACTCTTCTACCTCATAAGGAATATGTTACAATTAATTCATTAGGTAAGCA 128

||||||||||||||||||||||||||||||||||||||||||||||| ||||||||||||

Sbjct 10918 GGCTTCACTTACTCTTCTACCTCATAAGGAATATGTTACAATTAATTTATTAGGTAAGCA 10977

Query 129

TTTGTTTTATATTGGTTTTATTTCACCTGGGCTGAGATTTCAAGAAACA-CCCAGTCTTC 187

||||||||||||||||||||||||||||||||||||||||||||||||| ||||||||||

Sbjct 10978 TTTGTTTTATATTGGTTTTATTTCACCTGGGCTGAGATTTCAAGAAACACCCCAGTCTTC 11037

Query 188 ACAGTAACACATTTCACTAACACATTTACTAAACATCAGCAACTGTGGCCTGttaatttt 247

||||||||||||||||||||||||||||||||||||||||||||||||||||||||||||

Sbjct 11038 ACAGTAACACATTTCACTAACACATTTACTAAACATCAGCAACTGTGGCCTGTTAATTTT 11097

Query 248 tttaatagaaattttaagtcctcattttctttcggtgttttttaagcttaatttttCTGG 307

||||||||||||||||||||||||||||||||||||||||||||||||||||||||||||

Sbjct 11098 TTTAATAGAAATTTTAAGTCCTCATTTTCTTTCGGTGTTTTTTAAGCTTAATTTTTCTGG 11157

Query 308 CTTTATTCATAAATTCTTAAGGTCGACTACATTTGAAAAATCAAAGACCTGCATTTTAAA 367

|||||||||||||||||||||||| |||||||||||||||||||||||||||||||||||

Sbjct 11158 CTTTATTCATAAATTCTTAAGGTCAACTACATTTGAAAAATCAAAGACCTGCATTTTAAA 11217

Query 368 TTCTTATTCACCTCTGGCAAAACCATTCACAAACCATGGTAGTAAAGAGAAGGGTGACAC 427

||||||||||||||||||||||||||||||||||||||||||||||||||||||||||||

Sbjct 11218 TTCTTATTCACCTCTGGCAAAACCATTCACAAACCATGGTAGTAAAGAGAAGGGTGACAC 11277

Query 428 CTGGTGGCCATAGGTAAATGTACCACGGTGGTCCGGTGACCAGAGATGCAGCGCTGAGGG 487

||||||||||||||||||||||||||||||||||||||||||||||||||||||||||||

Sbjct 11278 CTGGTGGCCATAGGTAAATGTACCACGGTGGTCCGGTGACCAGAGATGCAGCGCTGAGGG 11337

Query 488 TTTTCCTGAAGGTAAAGGAATAAAGAATGGGTGGAGGGGCGTGCACTGGAAATCACTTGT 547

||||||||||||||||||||||||||||||||||||||||||||||||||||||||||||

Sbjct 11338 TTTTCCTGAAGGTAAAGGAATAAAGAATGGGTGGAGGGGCGTGCACTGGAAATCACTTGT 11397

Query 548 AGAGAAAAGCCCCTGAAAATTTGAGAAAACAAACAAGAAACTACTTACCAGCTATTTGAA 607

||||||||||||||||||||||||||||||||||||||||||||||||||||||||||||

Sbjct 11398 AGAGAAAAGCCCCTGAAAATTTGAGAAAACAAACAAGAAACTACTTACCAGCTATTTGAA 11457

Query 608 TTGCTGGAATCACAGGCCATTGCTGAGCTGCCTGAACTGGGAACACAACAGaaaaaaaaa 667

||||||||||||||||||||||||||||||||||||||||||||||||||||| ||||

Sbjct 11458 TTGCTGGAATCACAGGCCATTGCTGAGCTGCCTGAACTGGGAACACAACAGAAGGAAAAC 11517

Query 668 aaa 670

|||

Sbjct 11518 AAA 11520

**#9.**

GCTACCACTACTATCCGGCGAATTGGGCCCTCTAGAtGCATGCTCGAGCGGCCGCCAGTG

TGATGGATATCTGCAGAATTCGCCCTTAAGCAGTGGTATCAACGCAGAGTACTTTTTTTT

TTTTTTTTTTTTTTTTTTGTGTGTGAGTTTGTGAAGAAAGATTTATAAAATACTGACTGT

CTTATAAGATGAAATTATTATAATACAAACTAAGAATTTTGGCCTGTACGTCTCACCCAT

CCTCAATCAAGGGCGAATTCCATCCCACTGGCGGCCGTTACTAGTGGATCCGACCTCGGT

ACCAACCTTGGCGTAATCATGGTTGTAGCTGTTTCCTGTGTGAAAATGTTAGCCGCTCAC

CCTTCCCCCAACAATACAACCGGAAGCATA

Cloned fragment: 162 bp

The human HFE fragment is underlined and the cloning site is grey shaded

Alignment between the cloned sequence and the human HFE 3’UTR sequence:

None
